# Influence of Catalyst Composition on the Acidic Oxygen Evolution Reaction: From Single Oxide IrO_2_ to High-Entropy Oxide IrNiMnFeCoCuVO_x_

**DOI:** 10.3390/ma19071402

**Published:** 2026-03-31

**Authors:** Miguel Sánchez Martín, Miriam Alonso Menéndez, Daniel Barreda, Ricardo Santamaría, Clara Blanco, Victoria G. Rocha, Jonathan Ruiz Esquius

**Affiliations:** Instituto de Ciencia y Tecnología del Carbono (INCAR-CSIC), c/Francisco Pintado Fe 26, 33011 Oviedo, Spain; uo311007@uniovi.es (M.S.M.); miryalxaro@gmail.com (M.A.M.); daniel@incar.csic.es (D.B.);

**Keywords:** oxygen evolution reaction, proton exchange membrane electrolyser, high-entropy oxides, iridium oxide, green hydrogen

## Abstract

**Highlights:**

- Spinel oxides with up to seven metal elements were synthesised via a facile thermal decomposition method in air.
- Rutile IrO_2_ catalytic activity towards the acidic OER was improved by forming mixed metal oxides.
- Increasing the number of elements resulted in improved activity and stability up to MOx-5.
- Excessive metal elements resulted in a decrease in activity, possibly due to the dilution effect.
- Surface dissolution and reconstruction occurred during the reaction, even for high-entropy configurations.

**Abstract:**

Developing active and robust catalysts for the acidic oxygen evolution reaction (OER) with reduced Ir loading is still a challenge in the industrial production of green H_2_. In this work, several catalysts ranging from single metal oxides (e.g., IrO_2_) to high-entropy oxides (IrNiMnFeCoCuVO_x_) were synthesised through thermal decomposition in air to study the effect of the mixed-oxide composition in terms of activity and stability towards the acidic OER. Catalysts were named MOx-*n*, with *n* being the number of metal elements in the mixture. The results show that the activity of rutile IrO_2_ can be improved by introducing other elements into the composition. The best performance was obtained for MOx-4 to MOx-5, which delivered a current density of 10 mA cm^−2^ at an overpotential (*η*_10_) of 279 ± 4 mV; approx. 100 mV lower than IrO_2_ at a comparable Ir loading and with better stability. Nevertheless, further increasing the complexity of the mixed oxide resulted in an evident degradation in terms of activity and stability. It is worth noting that surface dissolution and reconstruction occurred with all mixed-oxide catalysts, including high-entropy configurations.

## 1. Introduction

Global technology is shifting towards electrification, with the electricity demand increasing faster than that for other means of energy generation [1]. With it, there has been a boom in renewable energy (RE) production and the deployment of water electrolysers (WEs) for the production of green hydrogen (H_2_) [2]. Hydrogen can be used as an C-free energy vector while allowing for the decarbonisation of the hard-to-abate industry [3]. In this context, the global production of green hydrogen is set to grow exponentially in the upcoming years [4]. However, in 2023, only 0.2% of the total H_2_ produced in Europe was obtained from the electrolysis of water [4].

Proton exchange membrane water electrolysers (PEM-WEs) have many advantages over other technologies, for instance, the production of high-purity H_2_ (99.99%) that is already pressurised (30 bar) for distribution; compact designs and high efficiency due to operating at high current densities (0.6–3.0 A cm^−2^); and fast response and easy plant balancing, which allows PEMs to be readily coupled with RE production [5,6]. Nevertheless, PEM-WEs require catalysts based on noble metals at the anode and at the cathode. The development of catalysts for the oxygen evolution reaction (OER), which occurs at the anode, is especially challenging due to the harsh oxidising conditions generated during operation, which results in fast catalyst degradation and prevents the widespread implementation of PEM-WEs. To date, only IrO_2_-based formulations possess high enough resistance to corrosion and good activity to be employed as efficient catalysts for the OER. In addition, iridium is one of the scarcest elements in the Earth’s crust [7,8]. For PEM-WEs to be competitive, the EU has set a goal of reducing the current iridium loading (1–2 mg cm^−2^) to 0.4 mg cm^−2^ by 2035 [8], whilst the US Department of Energy aims to ultimately reduce the loading down to 0.125 mg cm^−2^ [9]. This has increased interest in the design of catalysts with optimal Ir utilisation for the acidic OER [10,11,12].

Generally, iridium oxide (IrO_2_) catalysts can be divided into two broad categories, amorphous and crystalline. Amorphous iridium oxo-hydroxides, Ir(O)_x_(OH)_z_ or IrO_x_ for simplicity, outperform their crystalline counterparts in terms of activity due to their flexible structure [13], and the co-existence of Ir^3+/4+^ centres [14,15] and electrophilic O^−^ sites [16,17,18]. Moreover, the high activity of IrO_x_ normally results in faster degradation rates compared to rutile IrO_2,_ [19,20,21] even though active sites generated during the OER are comparable between amorphous and crystalline catalysts [21,22]. In both systems, lattice oxygen is involved in the OER mechanism, with an intermediate reaction that can evolve either towards oxygen evolution or Ir dissolution [19,23]. In rutile IrO_2_, only the outmost surface participates in the reaction, with little contribution from lattice oxygen sites. In contrast, in IrO_x_, a thicker region, including lattice sites, participates in the reaction mechanism, resulting in higher catalytic activity but at the expense of faster degradation [21,24].

A common strategy to improve the performance of IrO_2_ catalysts, and concomitantly reduce the loading of Ir, is the design of mixed oxides, as this allows for the adjustment of the electronic structure and the tunning of the binding strength of the reaction intermediates [10,25]. For instance, Strasser and co-workers extensively studied Ir-Ni combinations for the acidic OER [26]. Their work showed that the geometric catalytic activity of IrO_2_ could be improved eightfold with the addition of Ni. The boost in activity was associated to the surface dissolution of Ni species leading to the formation of a porous structure rich in IrOx species at the surface [27,28]. Nevertheless, gains in activity were accompanied by an increase in Ir dissolution [26].

The development of high-entropy oxides (HEOs) may, in principle, allow one to overcome the activity–stability trade-off encountered in catalysts for the acidic OER. HEOs are made of at least five metal elements, distributed homogeneously in a thermodynamically stable single phase. Furthermore, due to the multimetal composition the lattice is strained and atomic diffusion is hindered, which renders HEOs’ outstanding chemical and thermal stability. Moreover, the variety of adsorption sites and partially charged sites leads to synergistic effects [29,30,31,32]. For instance, Baek et al. synthesized several spinel oxides, from single to quinary combinations using the rapid sol-flame method. The catalytic activity and stability improved with the complexity of the oxide. Thus, the quinary oxide (CoFeNiCrMn) outperformed the quaternary oxides (CoFeNiCr, CoFeNiMn), which in turn outperformed the ternary metal oxide (CoFeNi), and so forth [33]. When calculating the binding energy of OER intermediates over more than one thousand sites, they found that active sites on HEO tend to adsorb reaction intermediates more strongly than low- and medium-entropy oxides, which resulted in improved activity [33]. A comparable trend in activity ((FeCoNiCuMn)_2_O_3_ > (FeCoNiCu)_2_O_3_ > (FeCoCu)_2_O_3_ > (CoCu)O > Co_3_O_4_)) was also observed towards the OER in both alkaline and saline media by Li et al. [34].

For the acidic OER, where Ir is currently indispensable, it is possible to complement the Ir catalytic properties (corrosion resistance and activity towards the acidic OER) with the high entropy benefits (lattice strain and synergy) by combing it with electrochemically active elements (e.g., Ru, Ni, Co, Fe, and V) [35] and potentially stable oxides (e.g., Ti, Mn, Fe, Cu, and Sn) [36]. As an example, rutile (RuIrFeCoCrO_2_) synthesised by the molten salt method by different groups yielded a current density of 10 mA cm^−2^ at low overpotential (*η*_10_ of ca. 260 mV) and promising stability [37,38]. Through density functional theory Zhang et al. established that Cr prevented Ru from leaching, whilst Co and Ir were responsible for the improved activity [38].

In our group, we studied the effect of temperature on IrMnFeCoNiOx HEOs with a spinel structure supported on carbon fibres synthesised via thermal decomposition in air. Low annealing temperature (<350 °C) resulted in an amorphous phase which was unstable in acid; however, upon crystallisation (annealing > 450 °C) a *η*_10_ of 290 ± 10 mV was achieved when tested towards the OER, which was lower than that of amorphous IrO_x_ (333 ± 9) and rutile IrO_2_ (393 ± 17) catalysts synthesized in an analogous way and with comparable Ir loading (0.4 mg cm^−2^). Furthermore, the crystalline IrMnFeCoNiOx catalyst showed no signs of activity decay during the OER for 70 h at 10 mA cm^−2^ [39].

Expanding from previous results, where annealing a mixture of metal chlorides at 500 °C resulted in the formation of a high-entropy spinel with improved catalytic activity towards the acidic OER [39], the aim of this work is to determine the effect of the elemental composition on OER catalysts. In this regard, the synthesis conditions were kept constant, but the number of elements in mixed oxides was progressively increased: Namely, IrOx, IrNiOx, IrNiMnOx, IrNiMnFeOx, IrNiMnFeCoOx, IrNiMnFeCoCuOx and IrNiMnFeCoCuVOx were prepared by thermal decomposition in air (500 °C, 1 h), while maintaining the Ir loading on the carbon fibres constant at ca. 0.4 mg cm^−2^. Hereafter, for simplicity catalysts will be labelled as MOx-*n*, *n* indicating the number of metal elements. For instance, IrOx and IrNiOx are labelled as MOx-1 and MOx-2, respectively. The results indicate that the catalytic activity of MOx-1 (*η*_10_ of 374 ± 5) improved progressively with the number of elements until MOx-4 (*η*_10_ of 279 ± 4). No additional beneficial effects in terms of activity or stability were observed by increasing the number of elements in the composition from MOx-4 up to MOx-6. Besides, further increasing the complexity of the oxide, as for MOx-7, resulted in a decrease in the catalytic activity and stability.

## 2. Materials and Methods

All chemicals employed in this work were used as received unless otherwise stated. Iridium(III) chloride hydrate (IrCl_3_·xH_2_O), manganese(II) chloride tetrahydrate (MnCl_2_·4H_2_O, 98%), iron(II) chloride tetrahydrate (FeCl_2_·4H_2_O, Supelco^®^, Bellefonte, PA, USA), nickel chloride (NiCl_2_, 98%), copper(II) chloride dihydrate (CuCl_2_·2H_2_O, 99%), vanadium(III) chloride (VCl_3_), perchloric acid (HClO_4_, 70%) and hydrochloric acid (HCl, 0.1 M, Supelco^®^, Bellefonte, PA, USA) were provided by Sigma-Aldrich (Burlington, MA, USA). Cobalt(II) chloride hexahydrate (CoCl_2_·6H_2_O) was supplied by Honeywell. Toray carbon paper (TGP-H-60, PTFE) was acquired from Thermo Scientific, Waltham, MA USA. Toray carbon paper (denoted hereafter as CFs) was heat-treated in air (5 °C min^−1^, 550 °C, 5 h, 100 mL min^−1^) prior to its use to remove the PTFE surface layer.

### 2.1. Synthesis of Materials

MOx-1 to MOx-7 were synthesised following an analogous methodology previously reported [39]. The concentration of Ir on the TCP substrate was maintained at 0.4 mg cm^−2^, whilst other metals were present in an equimolar concentration relative to Ir. Aqueous stock solutions (5 mL) of IrCl_3_ (0.1 M), NiCl_2_ (0.2 M in 0.1 M HCl) MnCl_2_ (0.2 M), FeCl_2_ (0.2 M in 0.1 M HCl) CoCl_2_ (0.2 M), CuCl_2_ (0.2 M), VCl_3_ (0.2 M) were prepared in advance.

As an illustrative example, the synthesis of MOx-7 (IrNiMnFeCoCuVOx) was as follows: 200 µL of IrCl_3_ and 100 µL of NiCl_2_, MnCl_2_, FeCl_2_, CoCl_2_, CuCl_2_ and VCl_3_ were mixed with an IKA Vortex (Wilmington, NC, USA) for 5 min. The stated volume served for the preparation of one aliquot; the volume to be added was adjusted accordingly to match the desired number of samples. To obtain an Ir loading of 0.4 mg cm^−2^, 214 µL from the mixed solution was added onto a 1 × 1 cm^−2^ area of the substrate. It is worth mentioning that the volume was drop-cast in six small additions, both sides of the substrate were coated equally, and that samples were dried (70 °C, 15 min, air) between additions. Finally, the samples were placed on an alumina crucible and annealed in a preheated muffle (500 °C, air). After 1 h, the crucible was removed and allowed to cool to room temperature.

### 2.2. Physico-Chemical Characterisation

X-Ray diffraction (XRD) patterns were recorded on a Bruker D8 using a CuK_α_ X-Ray source and equipped with a Göbel mirror; Hutchinson, MI, USA. To avoid slight variations and misinterpretation, patterns were adjusted to the main peak of the carbon substrate (002) at 26.41°. Raman spectroscopy was obtained on a Renishaw inVia Qontor spectrometer operating with a 532 nm DPSS laser, a Leica MD2700 x100 confocal microscope (Wetzlar, Germany) and a CCD detector. The equipment was calibrated prior to any acquisition using a silicon wafer at 520.80 cm^−1^. Scanning electron microscopy (SEM) was performed on a Quanta FEG 650 (FEI Company, Hillsboro, OR, USA) operating at 25 KV and equipped with an ETD detector and an Octane Elect Plus detector (EDAX Inc., Mahwah, NJ, USA) for dispersive energy spectroscopy (EDS). High-resolution scanning transmission electron microscopy (HRSTEM) images were obtained by the Laboratorio de Microscopias Avanzadas, within the University of Zaragoza, on a Titan (Thermofisher, Waltham, MA, USA) microscope with a CETCOR image corrector and a DCOR+ probe corrector operating at a voltage of 300 KV, coupled with a Thermofisher camera (Ceta, Waltham, MA, USA). EDS analysis was carried out with a SuperC G2 detector, San Francisco, CA, USA. X-Ray photoelectron spectroscopy (XPS) was acquired on a SPECS system operated at 10^−7^ Pa with a pass energy of 10 eV and equipped with a monochromatic Al K_α_ X-Ray source (175 W). Inductively coupled plasma mass spectrometry (ICP-MS) were performed on an Agilent 7700x (Agilent, Santa Clara, CA, USA).

### 2.3. Electrochemical Characterisation

To ensure reproducibility, at least three replicates from different preparations were tested; therefore, the results are presented as an average with the corresponding standard deviation, unless otherwise stated. Prior to electrochemical characterisation, the samples were washed in 15 mL of 0.1 M HClO_4_ under continuous stirring for 1 h, followed by copious rinsing with deionised water.

The samples were assessed towards the oxygen evolution reaction (OER) in a conventional three-electrode set up in 0.1 M HClO_4_ electrolyte without stirring. The samples were secured with a PTFE platinum electrode holder (Tailkuke, JJ110, Guangzhou, China) and used directly as the working electrode. A 2.5 × 2.5 cm platinum coated titanium mesh (RedoxMe, E-A-Pt/Ti) was employed as a counter electrode, and a hydrogen electrode (HydroFlex^®^, El Segundo CA, USA) was used as the reference. Counter, working and reference electrodes were placed in line, with a 1 cm gap between them.

The samples were stabilised by cyclic voltammetry (CV, 1.0 to 1.6 V_RHE_, 50 mV s^−1^, 100 cycles). The apparent activity was obtained by linear sweep voltammetry (LSV, 1.0 to 1.6 V_RHE_, 2 mV s^−1^), whilst stability was assessed by chronopotentiometry (CP, 10 mA cm^−2^, 12 h and CP at 10, 20, 30, 40 and 50 mA cm^−2^, for 8 h for each current density). Tafel slopes were obtained by LSV under quasi-stationary conditions (1.44 to 1.51 V_RHE_, 0.5 mV s^−1^). The electrochemically active surface area (ECSA) was obtained on a non-faradaic region by CV (0.02 to 0.12 V_RHE_) at various scan rates (2 to 100 mA s^−1^). The non-faradaic region was determined based on CV (0 to 1.4 V_RHE_, 5 mV s^−1^).

## 3. Results and Discussion

### 3.1. Physicochemical Characterisation

**Figure 1:**
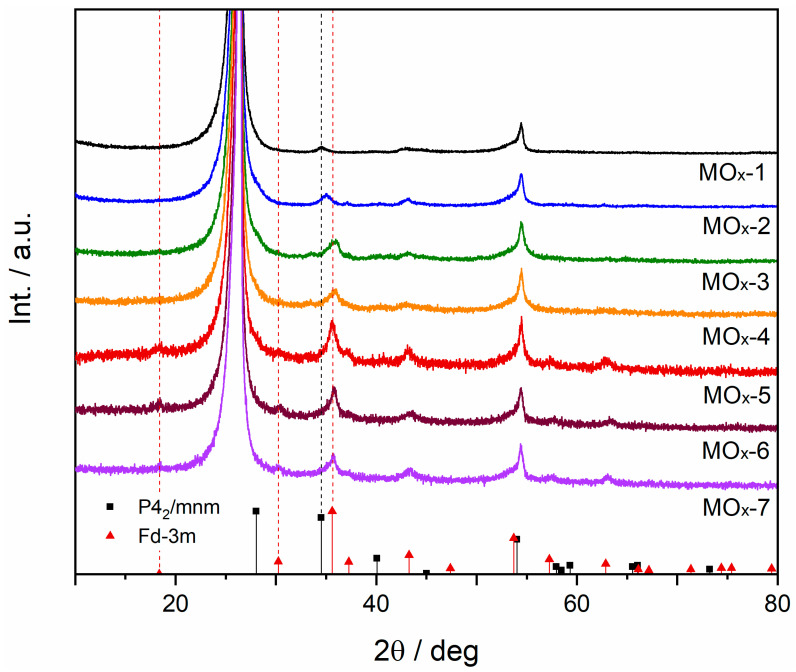
XRD patterns for MOx-*n* catalysts prepared by thermal decomposition of the corresponding metal chloride mixture.

MOx-*n* catalysts (namely IrOx, IrNiOx, IrNiMnOx, IrNiMnFeOx, IrNiMnFeCoOx, IrNiMnFeCoCuOx, and IrNiMnFeCoCuVOx) were synthesised over carbon fibres via thermal decomposition (550 °C, 1 h, air) of the corresponding mixture of metal chlorides. Metal loading onto the carbon substrate was confirmed by inductively coupled plasma mass spectrometry (ICP-MS) of mixed solutions: Ir 0.33 mg cm^−2^, Ni 0.13 mg cm^−2^, Mn 0.14 mg cm^−2^, Fe 0.14 mg cm^−2^, Co 0.16 mg cm^−2^, Cu 0.16 mg cm^−2^, and V 0.12 mg cm^−2^. To characterise the phases formed after annealing, samples were analysed by X-Ray diffraction (XRD, Figure 1). The main peaks at 26.6°, 42.9° and 54.4° correspond to diffraction peaks from the graphitic carbon fibres (CFs) of the substrate (Figure S1). As expected, the impregnation of the carbon substrate only with IrCl_3_ led to the crystallisation of rutile IrO_2_ (space group P4_2_/mnm, COD 2101854). It is worth mentioning that the main peak of rutile IrO_2_ at 28.1° (plane 100) is not observed as it overlaps with the main peak of the carbon substrate at 26.6°. The 101 plane of the P4_2_/mnm phase at 34.5° (MOx-1) shifted to 35.1° for MOx-2, suggesting that Ni atoms are incorporated into the lattice within the P4_2_/mnm phase. Moreover, extra peaks at 37.1° and 43.2° were assigned to a Fd-3m space group (e.g., spinel, COD 2300289). No peaks related to the P4_2_/mnm phase were observed after the addition of Mn, as for MOx-3, with the spinel Fd-3m phase being the main phase instead. As observed in Figure 1, a small peak at 33.4° assigned to the formation of Mn_2_O_3_ (space group Ia-3, plane 222, COD 1514106) was also detected, though the exact phase cannot be confirmed based on a single peak. Note that the combination of Mn only with Ni did not lead to the formation of multiple phases (Figure S2), but the combination of the three metal elements (Ir, Ni and Mn) resulted in phase segregation. For MOx-5 to MOx-7, peaks that correspond to the Mn_2_O_3_ secondary phase were not clearly identified, which could be due to either a decrease in the crystallite size or the incorporation of that phase into the main phase as a result of the formation of a high-entropy spinel oxide.

**Figure 2:**
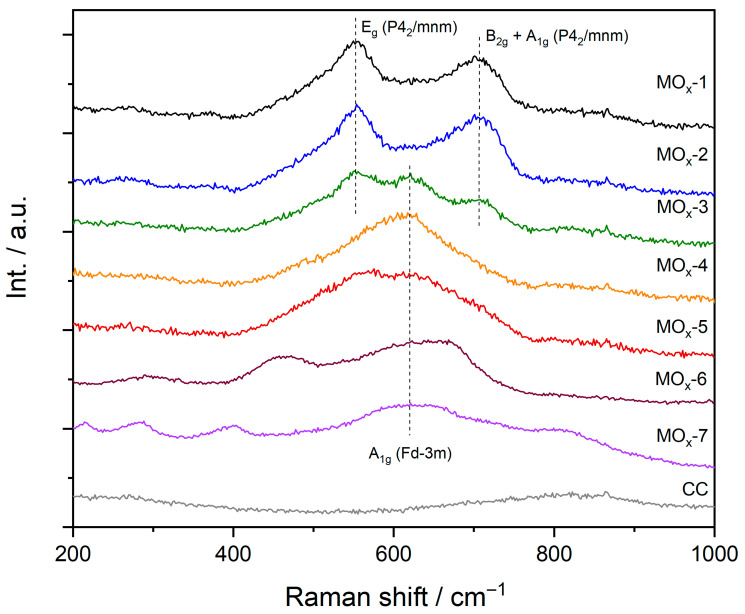
Raman spectra for MOx-*n* compared to the Raman spectra of the CFs substrate.

To further investigate the structure of the materials, MOx-*n* were also characterised by Raman spectroscopy (Figure 2). As expected, the E_g_ (ca. 552 cm^−1^) and B_2g_ + A_1g_ (ca. 706 cm^−1^) bands characteristic of rutile IrO_2_ [40] were observed for MOx-1. These rutile IrO_2_ bands were also well defined for MOx-2 and MOx-3 catalysts. Nevertheless, an extra band at ca. 620 cm^−1^ appeared for the MOx-3 sample, which could be assigned to the A_1g_ mode for cubic spinel oxides, as suggested by XRD. For MOx-4, only a broad and asymmetric peak was observed, which indicates that the local structure of the spinel is highly disordered (e.g., octahedral or tetrahedral distortions) and that cationic sites are occupied randomly by diverse cations (e.g., mixture of spinel, inverse spinel and/or partially inverted spinel) [41]. With increasing the number of cations within the structure, the Raman spectra progressively became more defined, indicating a higher degree of local ordering (e.g., non-distorted octahedral and tetrahedral distortions, and preference of elements for specific cationic sites in the spinel). For instance, the space group Fd-3m has five Raman active modes (A_1g_ +·E_g_ + 3F_2g_), though normally only three modes are clearly defined (A_1g_, F_2g_(2) and F_2g_(3), [42] as observed for MOx-6 and MOx-7, respectively.

**Figure 3:**
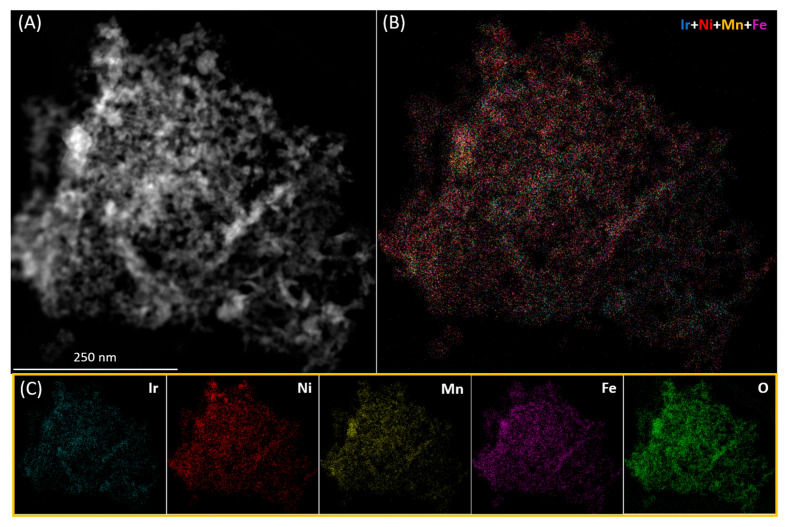
(**A**) STEM-EDS image for MOx-4, (**B**) combined elemental distribution and (**C**) elemental distribution of single elements throughout the material.

To determine the morphology and the elemental distribution at the nanoscale high-angle annular dark-field scanning transmission electron microscopy (HAADF-STEM) and energy dispersive X-Ray spectroscopy (STEM-EDS) were performed on MOx-*n* catalysts. Two different structures were encountered for MOx-1. The main structure is porous and made of small aggregates (7.9 ± 3.4 nm), whilst rods of 20–80 nm length intermixed with the main phase (Figure S3). As expected, STEM-EDS confirmed that the porous region is composed entirely of Ir and O. Nevertheless, Si was also detected in the rod-like structure (Figure S4), which may originate from contamination from the muffle oven’s lining employed during heat treatment. The elemental distribution on MOx-2 (Figure S5) and MOx-3 (Figure S6) is fairly homogeneous, with areas rich in either Ni or Mn, indicating that these materials do not form a single-phase solid solution. Increasing the number of metal elements in the mixture, as for MOx-4 and MOx-5, resulted in the homogeneous distribution of metal elements (Figure 3 and Figure S7), apart from the rod-like structures that are rich in Ir (Figures S8 and S9). Adding further elements to the mixture, as for MOx-6 and MOx-7, led to partial Ir segregation (Figures S10 and S11).

**Figure 4:**
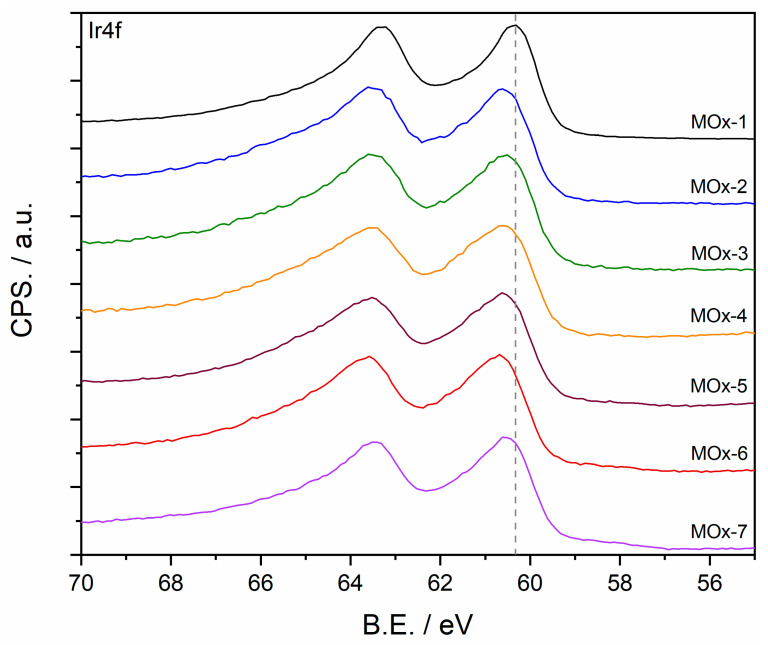
Ir4f peak for as prepared MOx-*n* catalysts. The dashed line represents the B.E. position for MOx-1.

X-Ray photoelectron spectroscopy (XPS) was employed to study chemical differences between catalysts at the surface level. Prior to analysis, all measured peaks were calibrated to the C1s peak at 284.5 eV (sp^2^ from the carbon substrate) [43]. The interpretation of the Ir4f peak is highly challenging due to spin–orbit coupling and electron correlation, in addition to peak overlapping with Ni3p, Ir5p1/2 and Mn3p peaks [43,44]. Therefore, the Ir4f peak interpretation was discussed based on the peak shape without any fitting (Figure 4). The Ir4f peak of MOx-1 was red-shifted by 0.2 eV compared to that of the mixed-oxide catalysts (MOx-2 to MOx-7); in addition, the Ir4f peak of MOx-1 was more asymmetric and narrower. These differences in the Ir4f peak suggest that MOx-1 is predominantly composed of Ir^4+^ sites, in agreement with XRD characterisation, whilst a mixture of Ir^3+^ and Ir^4+^ sites coexist on the mixed oxides [44,45]. Likewise, interpretation of the 2p peaks of transition metal oxides is also challenging due to peak overlapping, peak asymmetries, multiplet splitting, shake-up and plasmon loss structure [46,47]. Additionally, in this study, the chemical composition changes from sample to sample, which can also influence the peak shape. Therefore, Ni2p, Mn2p, Fe2p, Co2p, Cu2p, and V2p peak interpretation was based on peak position and shape (Figure S12). The binding energy (B.E.) of the Ni2p peak for the MOx-2 to MOx-7 catalysts was centred at 853.5–853.9 eV, closer to the B.E. of NiO; however, the peak shape resembles that of Ni(OH)_2_, possibly indicating the coexistence of NiO and Ni(OH)_2_. The Mn2p peak of the MOx-3 to MOx-5 catalysts were centred at 640.7 eV, which suggests that Mn sites were similar to those encountered in Mn_2_O_3_. In contrast, the Mn2p spectra for MOx-6 and MOx-7 showed a satellite feature at 647.4 eV, suggesting that the Mn sites were closer to MnO. A pronounced satellite peak at 716.3 eV in the Fe2p peak suggested the presence of FeO in the MOx-4 to MOx-7 catalysts. Similarly, a sharp satellite peak was also observed in the Co2p peak, indicating that the Co sites resemble those of CoO for the MOx-5 to MOx-7 catalysts. Similarly, the sharp satellite observed in the Cu2p peak indicates the presence of CuO [43,46]. It is worth mentioning that all peaks for the studied samples are shifted approximately by 2 eV compared to the B.E. reported for reference standards. For instance, the Ni2p peak of MOx-4 is centred at 853.5 eV, which is close to the B.E. reported for metallic Ni, but the peak shape is that of Ni(OH)_2_ reported at 855.6 eV [43]. This shift in B.E. may be attributed to the complexity of the composition and the interaction with other elements.

### 3.2. Electrochemical Characterisation

**Figure 5:**
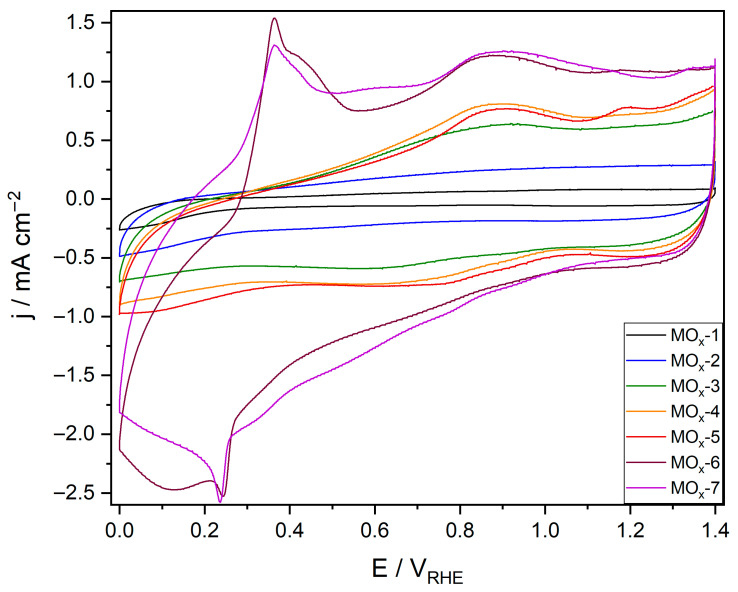
Redox transitions obtained by CV for MOx-*n* catalysts in 0.1 M HClO_4_.

Prior to electrochemical testing, catalysts were washed with acid (0.1 M HClO_4_, 15 mL, 1 h); the metal dissolution during this washing was quantified by ICP-MS. As seen in Table S1, the dissolution of Ir was negligible for MOx-1 and MOx-2 (i.e., less than 0.01% of the loaded Ir was detected by ICP-MS after acid washing). Furthermore, Ir dissolution increased with the number of elements, up to 0.31% for MOx-7. Ni, Mn, Fe, and Co dissolution remained comparable for the MOx-*n* catalysts (ca. 4–6%), whilst Cu and V dissolution was comparatively higher (ca. 11–16%) than that of the other metals. After acid washing, the MOx-*n* catalysts were assessed towards the acidic OER (0.1 M HClO_4_) in a conventional three-electrode set-up. It is worth noting that the MOx-*n* catalysts have an Ir loading of 0.4 mg cm^−2^, in line with the guidelines provided by the EU [8] and the US [9]. Readily corrodible species were removed from the surface of the material by cyclic voltammetry (CV, 1.0 to 1.6 V_RHE_, 50 mV s^−1^, and 100 cycles). The current density increased throughout the activation process for all catalysts, although more pronounced changes were progressively observed from MOx-1 to MOx-7 (Figure S13). After the activation process, a CV (0.0 to 1.4 V_RHE_, 5 mV s^−1^) was performed to determine the redox transitions occurring at the catalysts’ surface (Figure 5). No evident redox transitions were observed for MOx-1 and MOx-2, which exhibit a rectangular shape characteristic of rutile IrO_2_ [48]. For MOx-3 and MOx-4, a broad peak at ca. 0.9 V_RHE_, which may be attributed to the Ir^3+^/Ir^4+^ transition, was observed [49,50,51]. In addition, a peak at ca. 1.2 V_RHE_ that can be attributed to the Ir^4+^OH/Ir^4+δ^O redox transition was also observed for MOx-5 [49]. Two additional peaks at 0.36 V_RHE_ and 0.43 V_RHE_ were detected for MOx-6 and MOx-7, which cannot be assigned to the characteristic Ir-related redox transitions (e.g., Ir^3+^/Ir^4+^ at 0.9 V_RHE_ or Ir^4+^OH/Ir^4+δ^O at 1.2 V_RHE_). Since these additional transitions were observed upon the addition of Cu to the composition, it is reasonable to attribute these extra features to Cu-related redox transitions.

**Figure 6:**
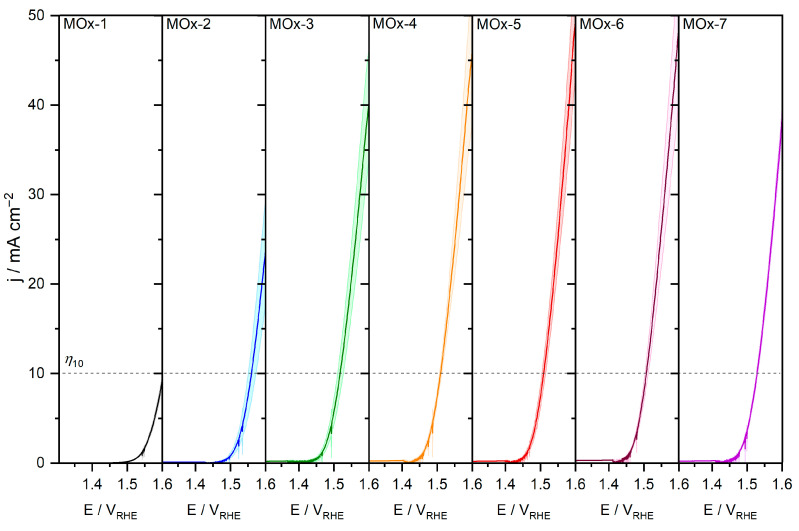
Apparent catalytic activity for MOx-*n* catalysts obtained by LSV in 0.1 M HClO_4_. Averaged activity is represented by solid lines, while standard deviation is shown as a light shading. Current density of 10 mA cm^−2^ is represented by a dashed line.

The apparent catalytic activity (normalised by the geometric area of 2 cm^2^) was obtained by linear sweep voltammetry (LSV, 1.0 to 1.6 V_RHE_, 2 mV s^−1^). As seen in Figure 6, the overpotential to reach a current density of 10 mA cm^−2^ (*η*_10_) decreased progressively with the addition of metal elements from 374 ± 5 mV for MOx-1 to 279 ± 4 mV for MOx-4. The apparent catalytic activity remained steady between MOx-4 and MOX-6, before decreasing to 298 ± 3 mV for MOx-7. The specific activity, obtained by normalizing the current density by the mass of the catalyst remaining on the carbon substrate after acid washing and after electrochemistry (Table S2), showed a similar trend to that observed for the apparent activity (Figure S14). Furthermore, at 1.5 V_RHE_, the specific activity improved significantly from 0.5 mA ${mg}_{cat}^{-1}$ for MOx-1 to 16.7 mA ${mg}_{cat}^{-1}$ for MOx-6, before declining to 7.9 mA ${mg}_{cat}^{-1}$ for MOx-7. The observed activity trend with the number of elements aligns with the discussion provided by Rossmeisl and co-workers for high-entropy alloys [52], whereby one metal in the mixture dominates the catalytic activity, in this case Ir. Hence, even though the activity can be improved by synergistic ligand interactions, the activity decreases with the complexity of the mixture due to dilution effects, that is, the concentration of active sites (e.g., Ir) at the surface decreases with the number of elements. In this scenario, low- to mid-entropy configurations outperform high-entropy compositions in terms of activity [52].

To assess whether changes in activity correlate with variations in the resistance of the system, electrochemical impedance spectroscopy (PEIS) was measured. The experimental data were fitted employing a model with three components [53,54] where Rs represents the ohmic resistance of the cell, R1 the resistance associated with the formation of an outermost hydrous layer, as reported for IrO_2_ catalysts [55], and R2 the resistance associated with the kinetics of the charge transfer reaction. The results (Figure S15, Table S3) indicate that R1 and R2 for MOx-1 decreased progressively with the addition of metals elements to the composition. However, the resistance associated with both charge transfer and the associated hydrous layer formation increases for MOx-7, in line with the observed decrease in the catalytic activity. Faster charge transfer Kinetics (R2) were encountered, particularly for MOx-4 and MOx-5, in agreement with their highest apparent activity.

Catalytic activity and other reaction metrics for the synthesized catalysts, such as Tafel slopes and the electrochemically active surface area (ECSA), can be found in Table 1. Catalytic metrics for the acidic OER of the synthesized catalysts compared against the top-performing catalysts reported in the literature are included in Table S4. It is worth mentioning that the majority of the high-entropy materials reported to date have been designed for the alkaline OER, with research on the acidic OER remaining relatively scarce [29,56].

Tafel slopes were obtained by LSV in quasi-stationary conditions (1.44 to 1.51 V_RHE_, 0.5 mV s^−1^) to study the effect of the catalysts’ composition on the reaction kinetics. As expected, the Tafel slope for MOx-1 (59.3 ± 1.2 mV dec^−1^) was in line with the values reported for rutile IrO_2_ (ca. 60 mV dec^−1^) [57,58]. The addition of Ni, as for MOx-2, promoted faster reaction kinetics, as indicated by the reduced Tafel slope (43.8 ± 2.8 mV dec^−1^). The value obtained for MOx-2 was in line with the values reported for amorphous iridium oxyhydroxides (ca. 40 mV dec^−1^) [59,60]. For iridium nickel mixed oxides previously reported in the literature, the majority of the nickel at the surface leaches during reaction, generating an amorphous surface enriched in hydroxyl groups that act as intermediates during the OER [26]. Comparable Tafel slope values were observed with an increasing number of elements in the catalyst composition, with MOx-5 (40.9 ± 0.5) and MOx-6 (40.5 ± 0.1) showing the fastest reaction kinetics. The obtained Tafel slopes, in addition to the post-characterization discussed later, suggest that surface leaching and reconstruction occur in the synthesized mixed oxides (MOx-2 to MOx-7), most likely driven by elements other than Ir, as commonly reported in the literature for Ir-containing mixed oxides [28,61,62,63,64,65].

To compare the extent of surface restructuring with the composition, the electrochemically active surface area (ECSA) for the MOx-*n* catalysts was determined by CV measurements in a non-Faradaic region (0.02 to 0.12 V_RHE_, 2–100 mV s^−1^; an illustrative example is provided in Figure S16 for MOx-4). It is worth highlighting that the obtained values are approximate since the carbon substrates also contribute to the ECSA values due to adsorption and desorption processes [66]. In addition, ECSA values depend on the reference specific capacitance (C_s_) employed. In this work, a C_s_ of 0.035 mF cm^−2^ commonly employed for acid electrolytes was adopted [67]. The ECSA value for MOx-1 (103 ± 4 cm^2^) almost quadrupled for MOx-2. Nevertheless, the overpotential of MOx-2 is only 45 mV lower than that of MOx-1. The poor correlation between ECSA and overpotential is due to the fact that the ECSA estimates the number of electrochemically active sites, but not all these sites are catalytically active towards the OER. Catalysts MOx-3 to MOx-6 exhibit comparable ECSA values (ca. 160 ± 20 cm^2^), as well as comparable apparent activity (ca. *η*_10_ of 280 mV). Interestingly, the ECSA for MOx-7 increased significantly compared to that of MOx-5, yet the apparent activity decreased.

**Figure 7:**
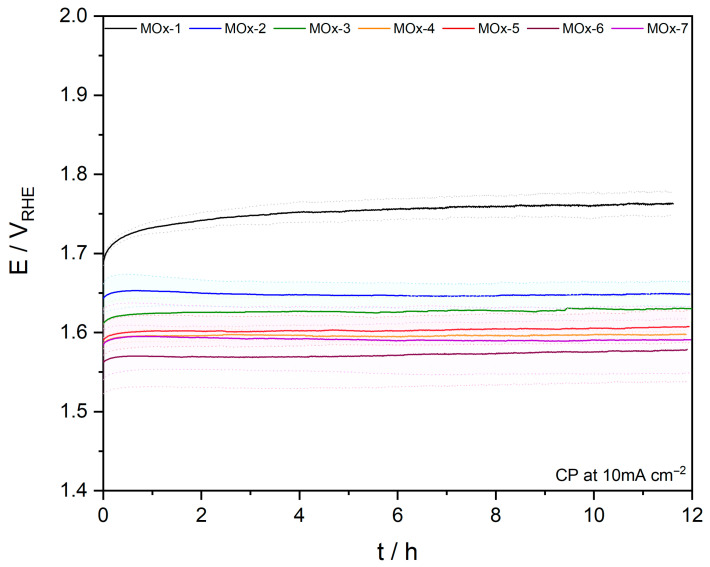
CP (10 mA cm^−2^, 10 h) obtained for MOx-*n* catalysts in 0.1 M HClO_4_. Averaged stability is represented by a solid line, whilst standard deviation is represented by the light shading within dashed lines.

**Figure 8:**
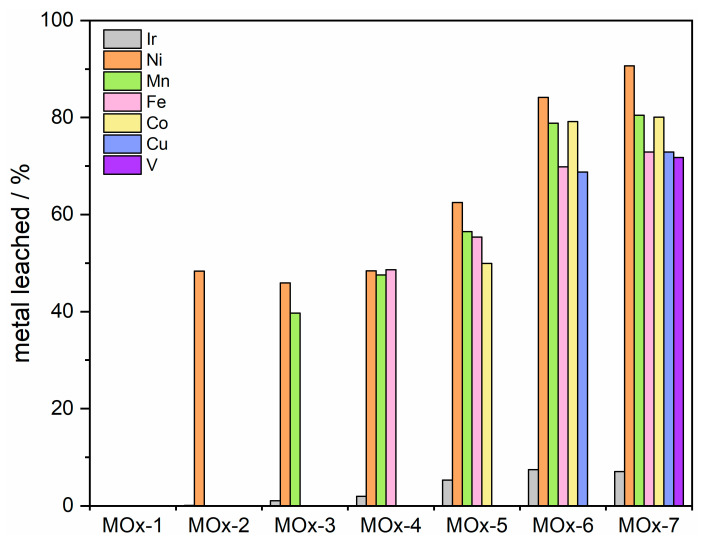
Metal leaching during electrochemistry as determined by ICP-MS.

The stability of the materials was assessed by chronopotentiometry (CP) at a current density of 10 mA cm^−2^ for 12 h. No evident activity degradation was observed for the MOx-2 to MOx-7 catalysts, with only MOx-1 showing a rise in potential (Figure 7). It is worth mentioning that the counter electrode turned reddish after the CP experiment when MOx-6 and MOx-7 were being tested, suggesting pronounced leaching. To further evaluate the stability of MOx-4 and MOx-5, a longer CP experiment (10 mA cm^−2^) was conducted for 150 h, during which only a ca. 29 mV increase in the potential was observed for both catalysts (Figure S17). This resulted in a small degradation rate of 0.2 mV h^−1^. To ensure that the apparent activity was maintained after CP (10 mA cm^−2^, 12 h), LSV was measured before and after CP to confirm that the apparent activity remained steady (Table 1). In line with the CP results, the overpotential to reach 10 mA cm^−2^ (*η*_10_) for MOx-1 increased by ca. 45 mV after the stability test. In contrast, no signs of deactivation were observed for the remaining catalysts. Electrochemical reactions, and particularly the acidic OER, due to the harsh oxidizing reaction conditions, often lead to surface reorganization and leaching [68]. To determine the amount of each metal corroded during the OER, the reaction electrolyte was analysed by ICP-MS (Figure 8, Table S2). For all elements, a higher leaching rate was observed with an increasing complexity of the composition. For instance, Ni leaching increased from 48.3% by mass for MOx-2 to 90.7% for MOx-7. The dissolution rates of Mn, Fe, Co, Cu, and V were comparable to that of Ni. The least corrosion was observed for Ir, although following a similar trend, with higher dissolution observed with an increasing complexity of the composition, only 2.0% of the initial iridium was leached for MOx-4. The rising dissolution rates of Ir with an increasing number of elements may be related to the greater formation of highly unsaturated Ir sites that are prone to corrosion due to the leaching of their surrounding elements. It is noteworthy that dissolution rates encompass the entire electrochemical characterization, including the CV activation step (1.0 to 1.6 V_RHE_, 50 mV s^−1^, 100 cycles); therefore, dissolution rates cannot be attributed merely to CP. Furthermore, the initial metal mass loading from the as-prepared samples was corrected for the dissolution observed after acid washing to obtain the dissolution during electrochemistry.

In addition, the stability of the materials was further assessed under dynamic conditions by CV (1.0–1.6 V_RHE_, 50 mV s^−1^, 2500 cycles). Three distinct tendencies were observed (Figure S18): (1) MOx-1 and MOx-7 lost activity until approximately the 1000th cycle, after which they stabilized for the remainder of the cycling stability test; (2) MOx-2, MOx-3, and MOx-6 showed consistently stable CV curves throughout all 2500 cycles; (3) MOx-4 and MOx-5 exhibited a sharp increase in the activity during the first 500 cycles, before stabilising for the remainder of the dynamic stability test.

### 3.3. Morphological and Chemical Changes During OER

**Figure 9:**
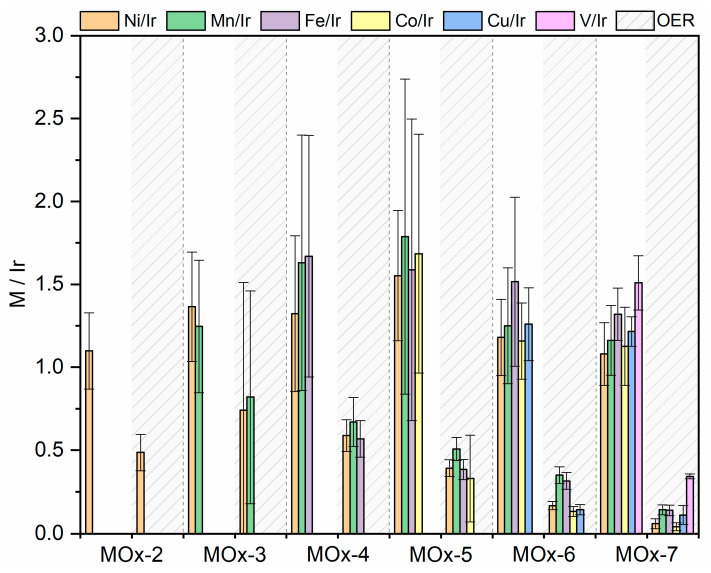
Element ratios normalized against Ir for each metal in MOx-*n* catalysts prior to (white area) and after CP at 10 mA cm^−2^ for 12 h (labelled as OER in the dashed grey area).

To assess the morphological and chemical changes after the OER (10 mA cm^−2^, 12 h, denoted hereafter as MOx-*n*_OER_), the samples were characterised by scanning electron microscopy–energy dispersive spectroscopy (SEM-EDS). It is worth mentioning that the loading of iridium was kept constant at 0.4 mg cm^−2^ for all catalysts, with the other metal elements introduced in an equimolar amount relative to Ir. Consequently, the layers covering the substrate are thicker for MOx-7 than for MOx-1. In general, the SEM images showed no significant changes in morphology after the OER (Figures S19–S24); however, MOx-7 showed pronounced agglomeration and a change in morphology (Figure S25). Nonetheless, EDS indicated that the elemental composition changed during the reaction (Figure 9), as previously confirmed by ICP-MS. It is worth noting that the M/Ir ratio (M = Ni, Mn, Fe, Co, Cu, and V) decreased for all catalysts and all metals, indicating that during the OER, elements other than Ir are prone to dissolution during the acidic OER, potentially forming a core–shell structure with an Ir-enriched outermost protective layer, as commonly reported for analogous Ir-containing mixed oxides [28,61,62,63,64].

**Figure 10:**
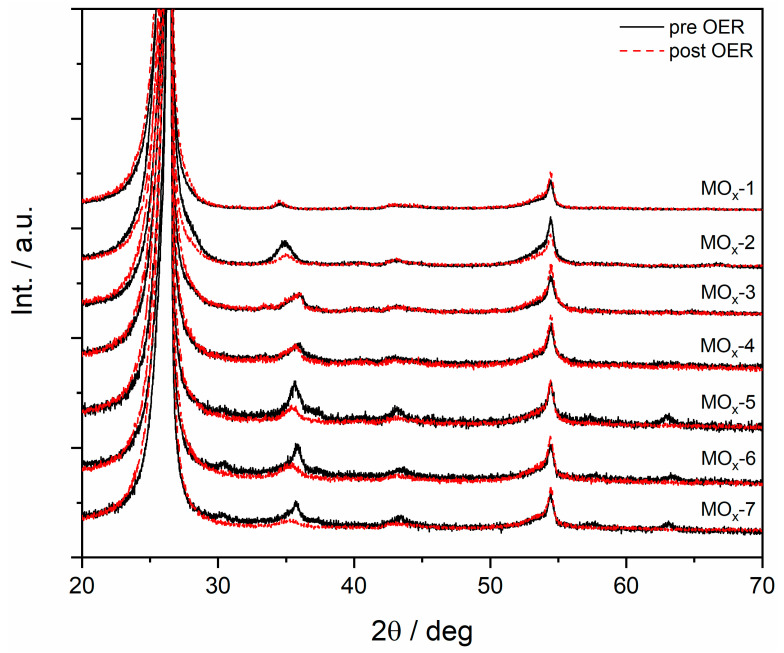
XRD patterns for MOx-*n* catalysts prior and post OER (10 mA cm^−2^, 12 h).

The XRD patterns of the materials before and after OER (10 mA cm^−2^, 12 h) were compared to study whether changes in composition were confined to the surface or extended into the bulk. As seen in Figure 10, no significant changes were observed for MOx-1, MOx-3, and MOx-4, indicating that no significant changes in the bulk structure had occurred. Nonetheless, the peaks became broader and less intense for MOx-2, MOx-5, MOx-6, and MOx-7, indicating structural changes affecting the bulk, although these did not result in a loss of activity under the explored conditions.

## 4. Conclusions

In summary, the design of mixed metal oxides is beneficial for improving the catalytic properties of rutile IrO_2_. The addition of elements to the catalyst composition enables the improvement of the apparent and the specific activity of MOx-1. Nevertheless, the dissolution of Ir also increased with the incorporation of additional metals into the composition, likely due to the high dissolution rates of leaching of Ni, Mn, Fe, Co, Cu, and V and the concomitant formation of coordinatively undersaturated Ir sites prone to corrosion.

The results obtained in this study indicate that a compromise between improved activity and stability can be achieved with medium entropy materials (e.g., MOx-4 and MOx-5). Further increasing the complexity of the mixed oxide (e.g., MOx-7) has a counterproductive effect, causing a drop in activity due to the dilution effect [52] and accelerated degradation. Therefore, the materials’ properties cannot be improved linearly with an increasing number of elements in the mixture.

It is worth emphasising that due to the experimental limitations, in conjunction with the vast area to explore in such complex systems, it was not possible in this work to attribute the improved activity entirely to the complexity of the mixed oxide, as different composition variations were not considered. For instance, in this study, MOx-2 was composed of IrNiOx, but other binary compositions are possible (e.g., IrMnOx, IrFeOx, and IrVOx).

## Figures and Tables

**Table 1:** Catalytic metrics, including apparent catalytic activity, Tafel slope, ECSA, apparent catalytic activity after chronopotentiometry (* CP, 10 mA cm^−2^ for 12 h), and specific activity for MOx-*n* catalysts.

Cat.	*η*_10_/mV	Tafel	ECSA/cm^2^	*η*_10_ after CP *	Specific Activity
MO_x_-1	374 ± 5	59.3 ± 1.2	103 ± 4	420 ± 32	0.5 mA ${mg}_{cat}^{-1}$
MO_x_-2	329 ± 13	43.8 ± 2.8	379 ± 107	309 ± 3.3	2.3 ${mA\;mg}_{cat}^{-1}$
MO_x_-3	288 ± 9	42.6 ± 0.8	153 ± 19	282 ± 2.9	11.8 ${mA\;mg}_{cat}^{-1}$
MO_x_-4	279 ± 4	43.8 ± 1.9	170 ± 24	270 ± 2.1	14.1 ${mA\;mg}_{cat}^{-1}$
MO_x_-5	279 ± 4	40.9 ± 0.5	160 ± 20	274 ± 2.3	13.5 ${mA\;mg}_{cat}^{-1}$
MO_x_-6	277 ± 4	40.5 ± 0.1	180 ± 24	270 ± 2.9	16.7 ${mA\;mg}_{cat}^{-1}$
MO_x_-7	298 ± 3	42.8 ± 0.4	294 ± 12	285 ± 2.2	7.9 ${mA\;mg}_{cat}^{-1}$

## Data Availability

The original contributions presented in this study are included in the article/Supplementary Materials. Further inquiries can be directed to the corresponding authors.

## Supplementary Materials

The following supporting information can be downloaded at: https://www.mdpi.com/article/10.3390/ma19071402/s1, Figure S1: XRD pattern for carbon fibres employed as substrate after annealing in air at 550 °C; Figure S2: XRD pattern for a NiMnOx synthesised for comparison; Figure S3: HAADF-STEM for MOx-1; Figure S4: STEM-EDS in different regions of MOx-1; Figure S5: HAADF-STEM and elemental mapping obtained by STEM-EDS for MOx-2; Figure S6: HAADF-STEM and elemental mapping obtained by STEM-EDS for MOx-3; Figure S7: HAADF-STEM and elemental mapping obtained by STEM-EDS for MOx-5; Figure S8: HAADF-STEM and elemental mapping obtained by STEM-EDS for rod-like structures encountered on MOx-4; Figure S9: HAADF-STEM and elemental mapping obtained by STEM-EDS for rod-like structures encountered on MOx-5; Figure S10: HAADF-STEM and elemental mapping obtained by STEM-EDS for MOx-6; Figure S11: HAADF-STEM and elemental mapping obtained by STEM-EDS for MOx-7; Figure S12: XPS 2p peaks for Ni, Mn, Fe, Co, Cu and V for MOx-2 to MOx-7; Table S1: Metal dissolution after washing MOx-n catalysts with HClO4 0.1 M (15 mL, 1 h); Figure S13: Cyclic voltammetry activation curves for (a) MOx-1, (b) MOx-2, (c) MOx-3, (d) MOx-4, (e) MOx-5, (f) MOx-6, and (g) MOx-7; Table S2: Metal loading for as prepared MOx-n catalysts, as well as metal loading for MOx-n catalysts after acid washing and electrochemistry (CV, 1.0 to 1.6 VRHE, 50 mV s^−1^, 100 cycles followed by CP 10 mA cm^−2^, 12 h). The loading for as prepared samples is based on ICP-MS analysis of the mixed solutions employed for the synthesis. Whereas the loading on the carbon substrate after acid washing and electrochemistry were derived by subtract-ing the as prepared loading to metals leaching obtained by analyzing the corresponding electrolyte by ICP-MS; Figure S14: Specific activity for MOx-n catalysts; Figure S15: Nyquists plots for MOx-*n* catalysts; Table S3: Parameters obtained from fitting the Nyquists plots employing the circuit represented in Figure S14; Table S4: Catalytic metric (geometric activity, reported stability, noble metal loading and Tafel slopes) of di-verse catalysts towards the acidic OER [69,70,71,72,73,74,75,76,77]. CF refers to carbon felt, CNT refers to carbon nanotubes, and CFs to carbon fibres; Figure S16: (a) CV performed on a non-Faradaic region for MOx-4 and (b) derived double layer capacitance (C_DL_) for the three replicates; Figure S17: Long-term stability test (10 mA cm^−2^, 150 h) for MOx-4 and MOx-5; Figure S18: Stability of MOx-*n* assessed under dynamic conditions by CV (1.0–1.6 V_RHE_, 50 mV s^−1^, 2500 cycles); Figure S19: SEM-EDS images for MOx-1 prior and after OER; Figure S20: SEM-EDS images for MOx-2 prior and after OER; Figure S21: SEM-EDS images for MOx-3 prior and after OER; Figure S22: SEM-EDS images for MOx-4 prior and after OER; Figure S23: SEM-EDS images for MOx-5 prior and after OER; Figure S24: SEM-EDS images for MOx-6 prior and after OER; Figure S25: SEM-EDS images for MOx-7 prior and after OER.

## Abbreviations

The following abbreviations are used in this manuscript:

AWE	Alkaline water electrolyser
COD	Crystallographic open database
CP	chronopotentiometry
CV	Cyclic voltammetry
HEO	High-entropy oxide
HER	Hydrogen evolution reaction
LSV	Linear sweep voltammetry
OER	Oxygen evolution reaction
PEM	Proton exchange membrane
RE	Renewable energy
WE	Water electrolyser
