# Supplementary material for: Influence of Catalyst Composition on the Acidic Oxygen Evolution Reaction: From Single Oxide IrO2 to High-Entropy Oxide IrNiMnFeCoCuVOx
Source: Materials (Basel). 2026 Mar 31;19(7):1402. doi: 10.3390/ma19071402 (PMC13074580; doi:10.3390/ma19071402)
Supplement: Supplementary file 1 [file materials-19-01402-s001.zip › materials-4190235-supplementary.pdf]

materials
MDPI

Supplementary Materials

Influence of Catalyst Composition on the Acidic Oxygen Evolution Reaction: From Single Oxide IrO_2 to High-Entropy Oxide $IrNiMnFeCoCuVO_x$

Miguel Sánchez Martín, Miriam Alonso Menéndez, Daniel Barreda, Ricardo Santamaría, Clara Blanco, Victoria G. Rocha * and Jonathan Ruiz Esquius *

Instituto de Ciencia y Tecnología del Carbono (INCAR-CSIC), c/Francisco Pintado Fe 26, 33011 Oviedo, Spain
* Correspondence: vgarciarocha@incar.csic.es (V.G.R.); jonathan.esquius@incar.csic.es (J.R.E.)

Supplementary A. Supplementary data.

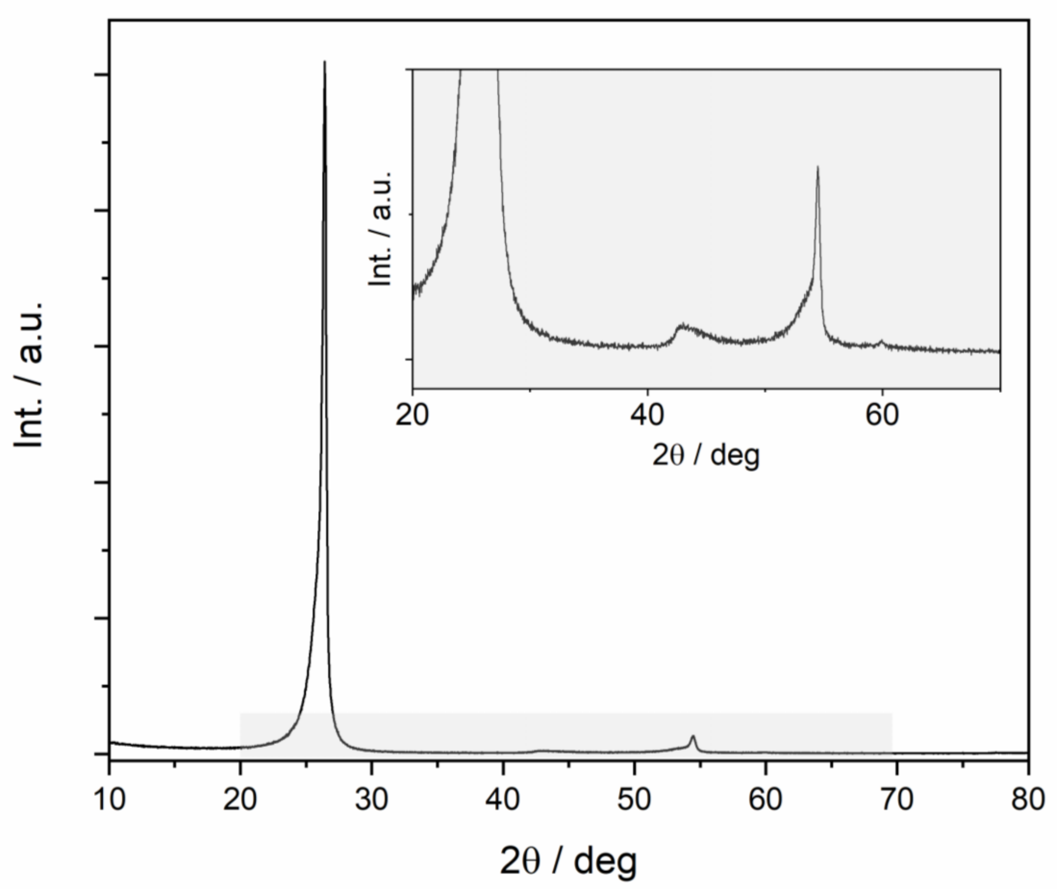

Figure S1. XRD pattern for carbon fibres employed as substrate after annealing in air at 550 ºC. The insert represents the area highlighted in grey in the main pattern.

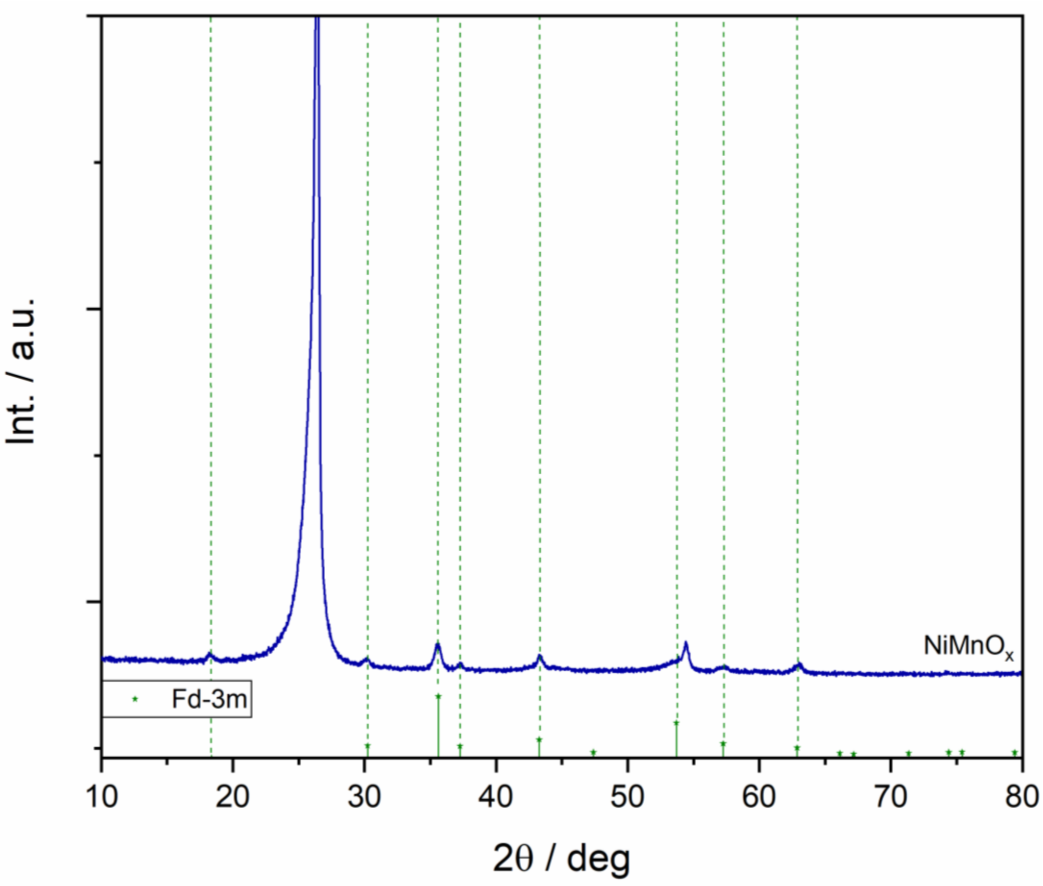

Figure S2. XRD pattern for a NiMnOx synthesised for comparison.

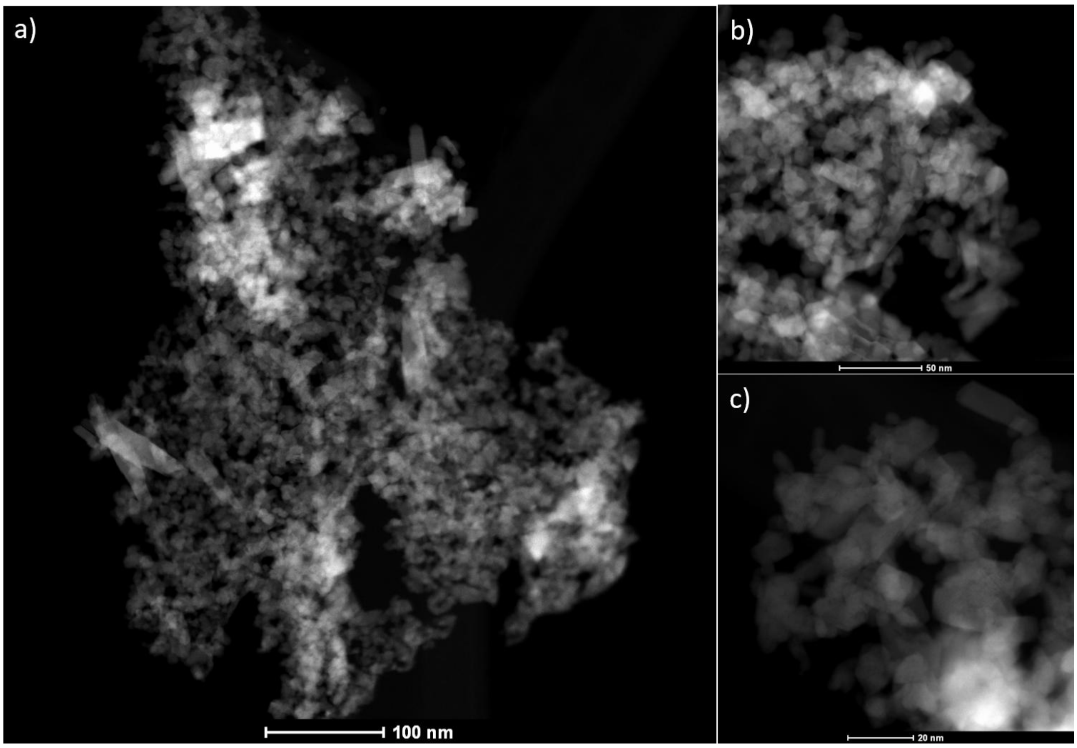

Figure S3. (**a**–**c**) HAADF-STEM images at different magnifications for MOx-1.

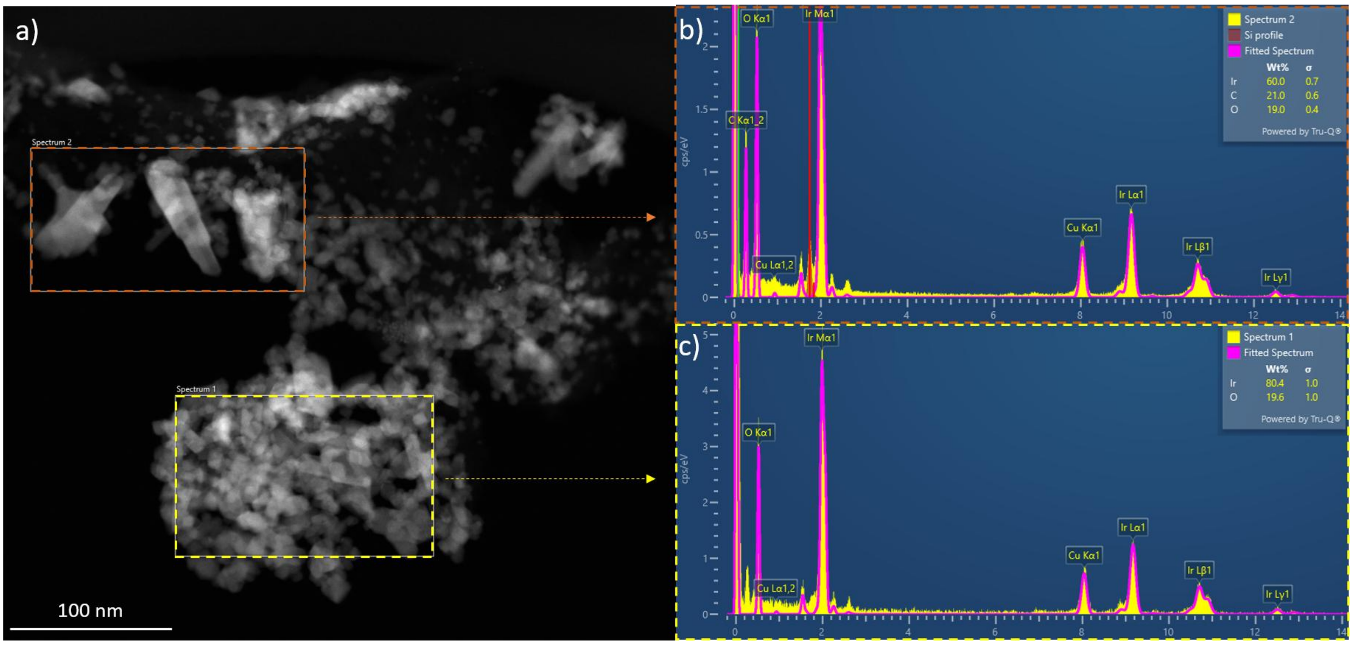

Figure S4. (**a**) STEM-EDS image for MOx-1 and EDS spectra in the area within (**b**) the red and (**c**) the yellow square in image (**a**).

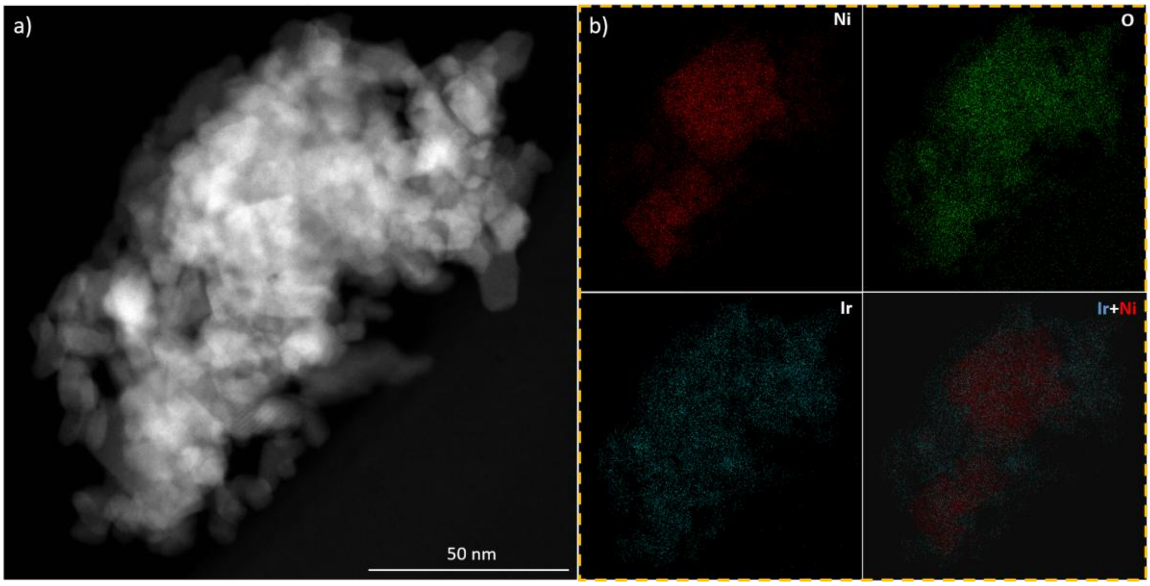

Figure S5. (**a**) HAADF-STEM image for MOx-2 and (**b**) elemental mapping obtained by STEM-EDS from image (**a**).

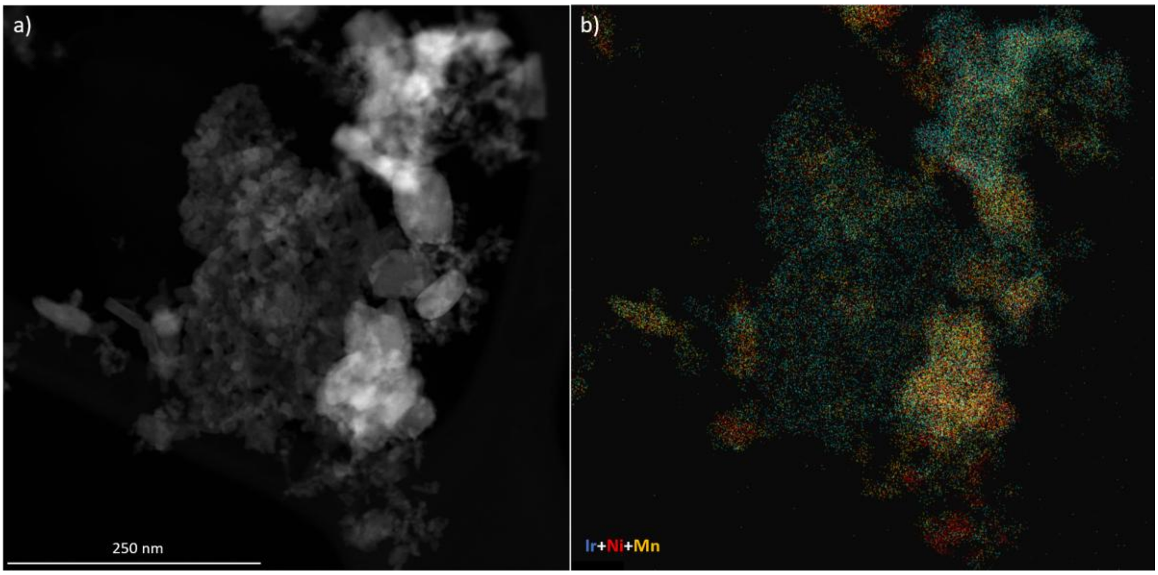

Figure S6. (**a**) HAADF-STEM image for MOx-3 and (**b**) elemental mapping obtained by STEM-EDS from image (**a**).

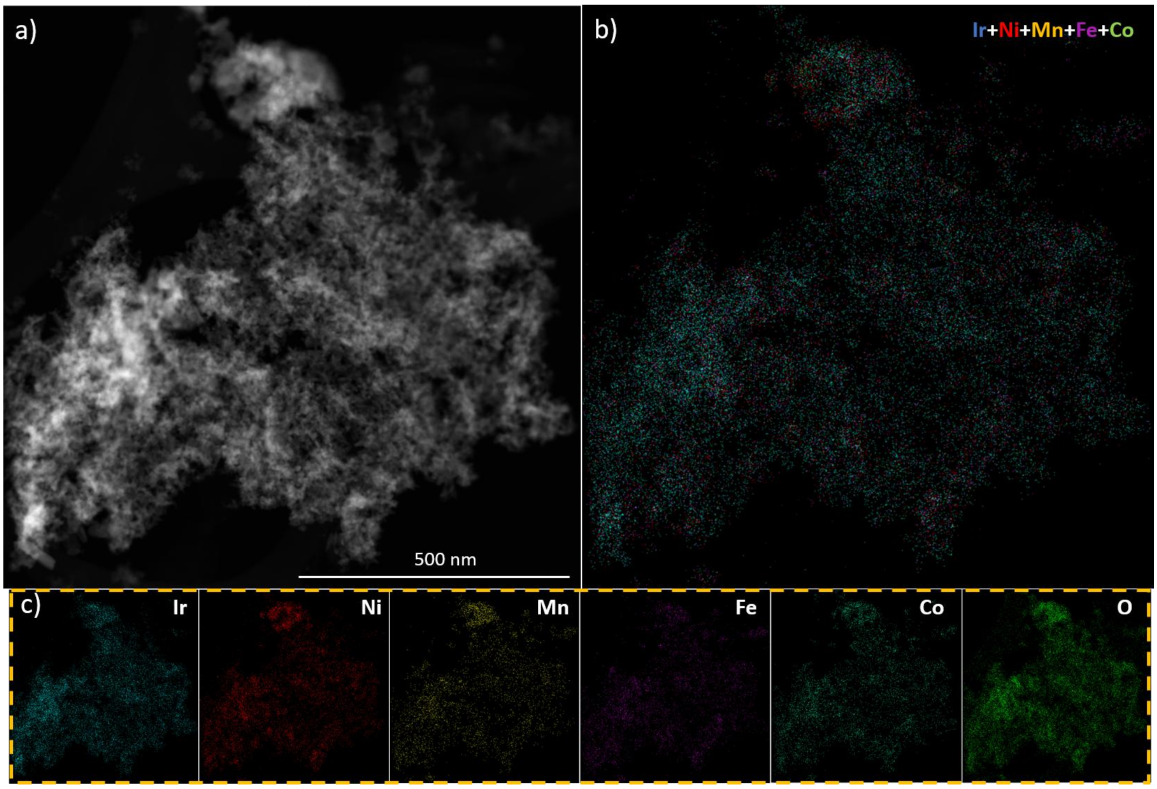

Figure S7. (**a**) HAADF-STEM image of MOx-5, (**b**) combined elemental mapping and (**c**) individual elemental mapping obtained by STEM-EDS from image (**a**).

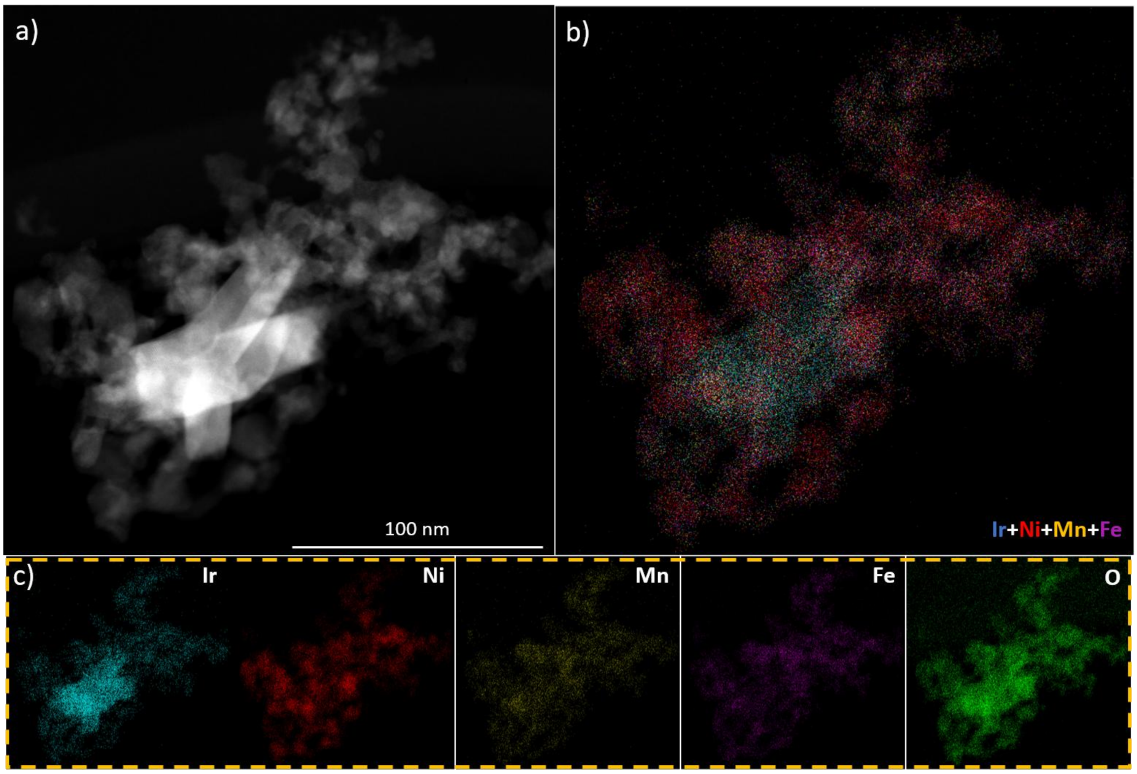

Figure S8. (**a**) HAADF-STEM image of rod-like structures encountered in MOx-4, (**b**) combined elemental mapping and (**c**) individual elemental mapping obtained by STEM-EDS from image (**a**).

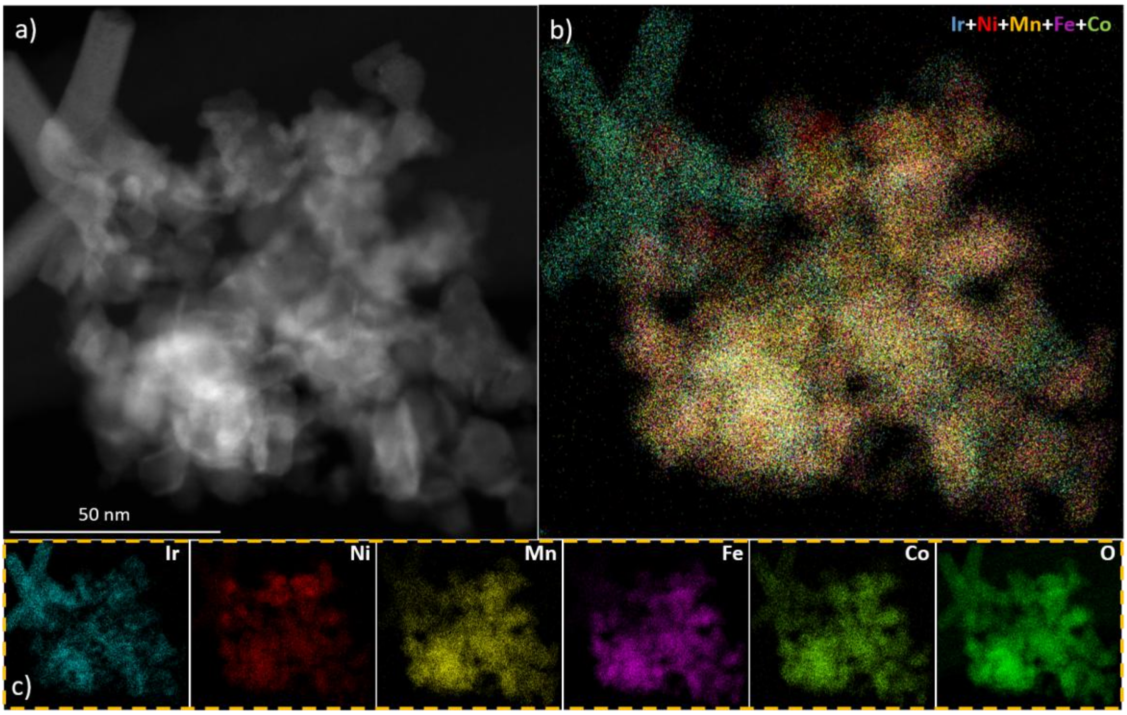

Figure S9. (**a**) HAADF-STEM image of rod-like structures encountered in MOx-5, (**b**) combined elemental mapping and (**c**) individual elemental mapping obtained by STEM-EDS from image (**a**).

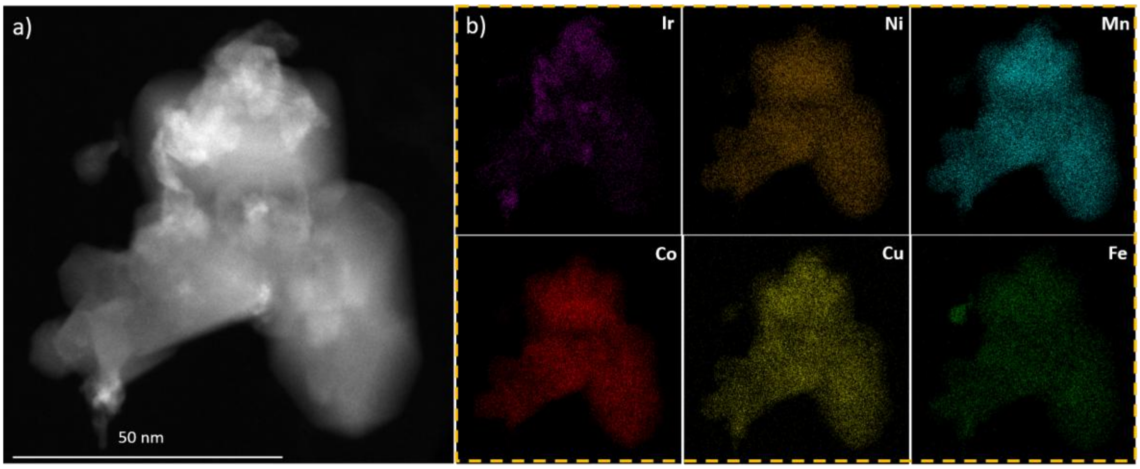

Figure S10. (**a**) HAADF-STEM image of MOx-6 and (**b**) elemental mapping obtained by STEM-EDS from image (**a**).

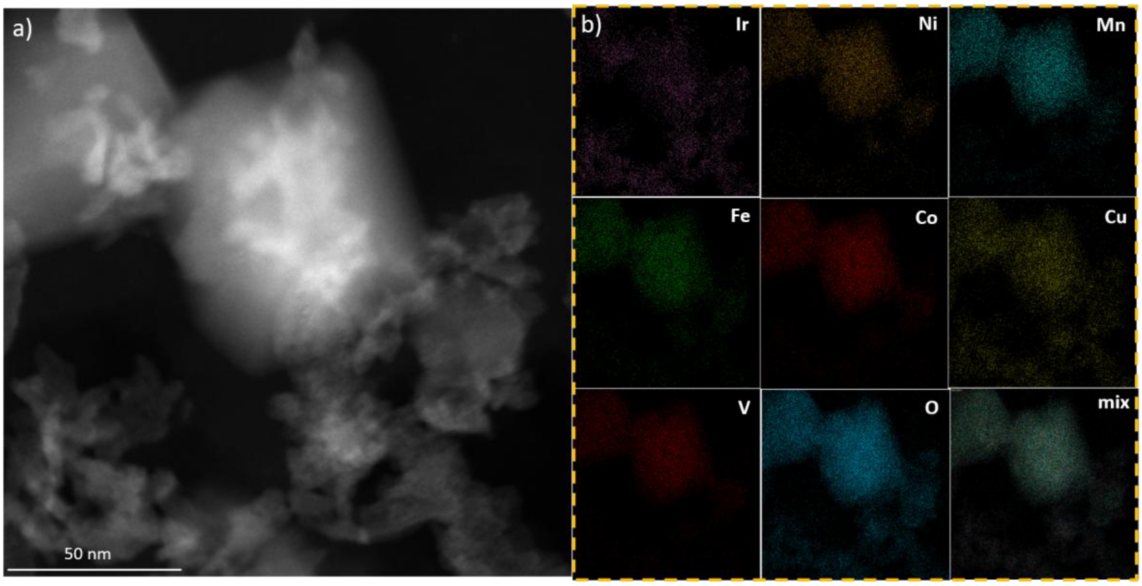

Figure S11. (**a**) HAADF-STEM image of MOx-7 and (**b**) elemental mapping obtained by STEM-EDS from image (**a**).

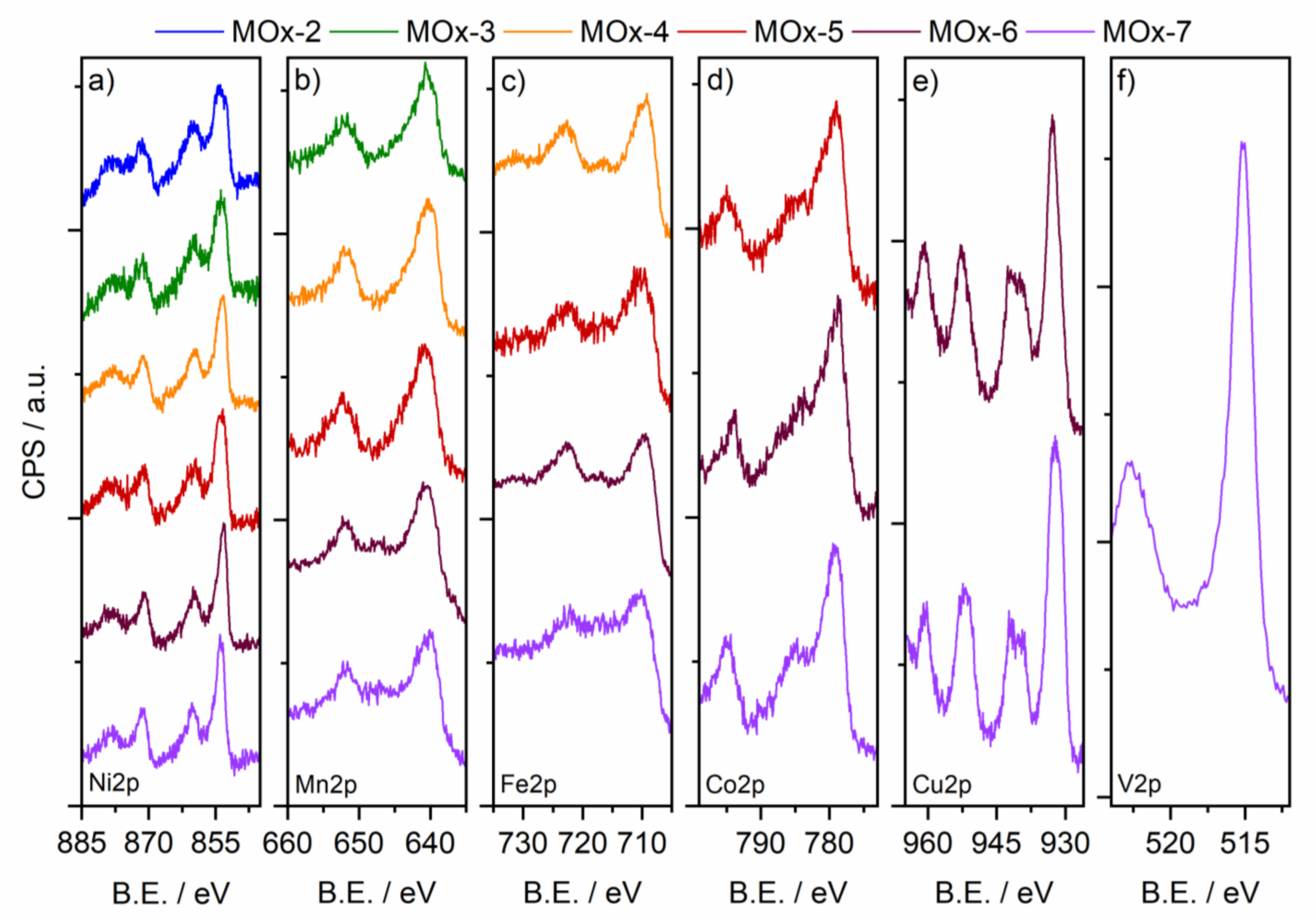

Figure S12. (**a**) Ni2p, (**b**) Mn2p, (**c**) Fe2p, (**d**) Co2p, (**e**) Cu2p and (**f**) V2p peaks obtained by XPS for MOx-2 to MOx-7 catalysts.

Table S1. Metal dissolution after washing MOx-*n* catalysts with HClO4 0.1 M (15 mL, 1 h).

	**mg leached (x 1/1000)**							**Dissolution / %**						
**Cat.**	Ir	Ni	Mn	Fe	Co	Cu	V	Ir	Ni	Mn	Fe	Co	Cu	V
MOx-1	0.02							< 0.01						
MOx-2	0.04	8.9						< 0.01	3.19					
MOx-3	0.2	11.8	14.3					0.04	4.25	5.14				
MOx-4	0.5	11.6	18.8	10.4				0.09	4.17	6.79	3.77			
MOx-5	0.6	8.4	14.0	6.6	1.7			0.09	3.04	5.05	2.38	5.43		
MOx-6	0.9	3.9	8.4	5.7	8.6	49.0		0.15	1.45	3.08	2.02	2.74	15.28	
MOx-7	1.9	12.5	17.0	11.6	24.6	50.8	28.2	0.31	4.56	6.22	4.02	7.88	15.93	11.61

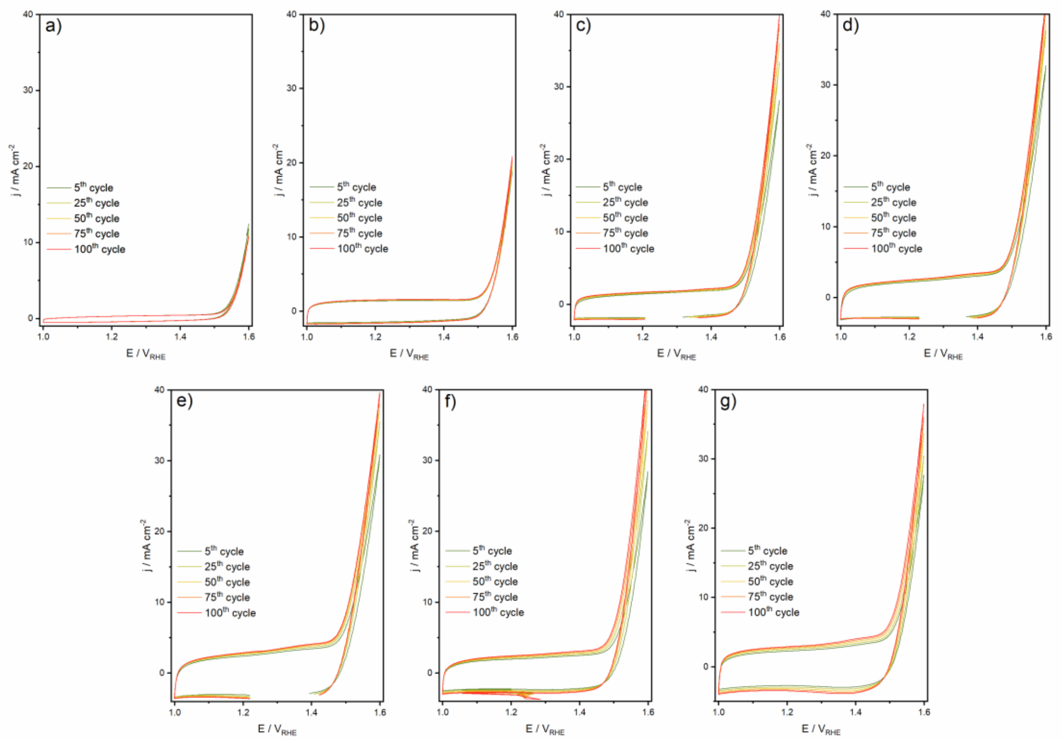

Figure S13. Cyclic voltammetry activation curves for (**a**) MOx-1, (**b**) MOx-2, (**c**) MOx-3, (**d**) MOx-4, (**e**) MOx-5, (**f**) MOx-6, and (**g**) MOx-7.

Table S2. Metal loading for as prepared MOx-*n* catalysts, as well as metal loading for MOx-*n* catalysts after acid washing and electrochemistry (CV, 1.0 to 1.6 V_{RHE}, 50 mV s^{-1}, 100 cycles followed by CP 10 mA cm-2, 12 h). The loading for as prepared samples is based on ICP-MS analysis of the mixed solutions employed for the synthesis. Whereas the loading on the carbon substrate after acid washing and electrochemistry were derived by subtracting the as prepared loading to metals leaching obtained by analyzing the corresponding electrolyte by ICP-MS.

	**As prepared (mg cm^{-2})**								**After acid washing (mg cm^{-2})**								**After electrochemistry (mg cm^{-2})**							
	Ir	Ni	Mn	Fe	Co	Cu	V	tot	Ir	Ni	Mn	Fe	Co	Cu	V	tot	Ir	Ni	Mn	Fe	Co	Cu	V	tot
**MOx-1**	0.33							0.33	0.33							0.33	0.33							0.33
**MOx-2**	0.33	0.14						0.47	0.33	0.13						0.46	0.33	0.07						0.40
**MOx-3**	0.33	0.14	0.14					0.61	0.33	0.13	0.13					0.59	0.32	0.07	0.08					0.48
**MOx-4**	0.33	0.14	0.14	0.14				0.75	0.33	0.13	0.13	0.13				0.73	0.32	0.07	0.07	0.07				0.52
**MOx-5**	0.33	0.14	0.14	0.14	0.16			0.90	0.33	0.13	0.13	0.13	0.15			0.88	0.31	0.05	0.05	0.06	0.08			0.55
**MOx-6**	0.32	0.14	0.14	0.14	0.15	0.16		1.05	0.32	0.13	0.13	0.14	0.15	0.14		1.01	0.30	0.02	0.03	0.04	0.03	0.04		0.46
**MOx-7**	0.33	0.14	0.14	0.14	0.15	0.16	0.12	1.18	0.32	0.13	0.13	0.14	0.15	0.13	0.11	1.10	0.30	0.01	0.02	0.04	0.03	0.04	0.03	0.47

https://doi.org/10.3390/xxxxx

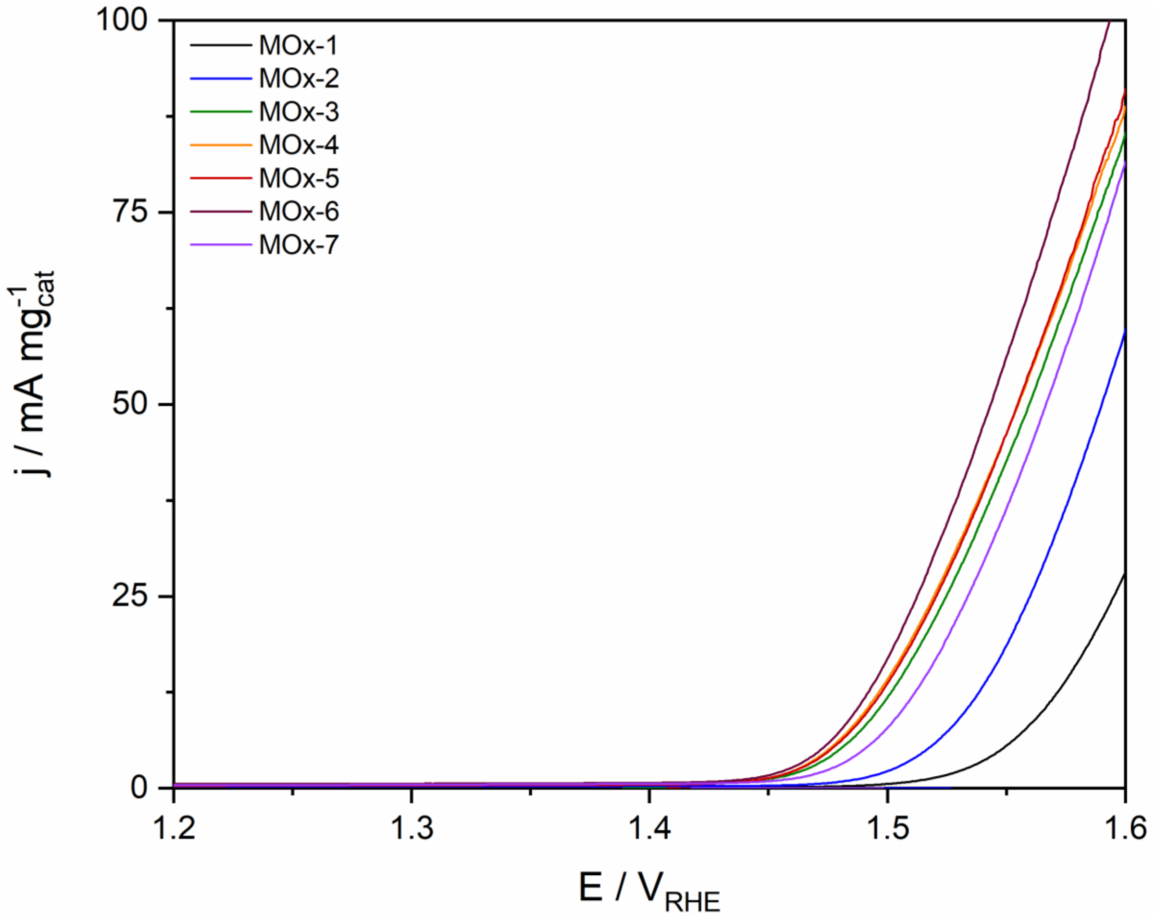

Figure S14. Specific activity obtained by dividing the current intensity by the catalysts mass remaining after acid washing and electrochemistry.

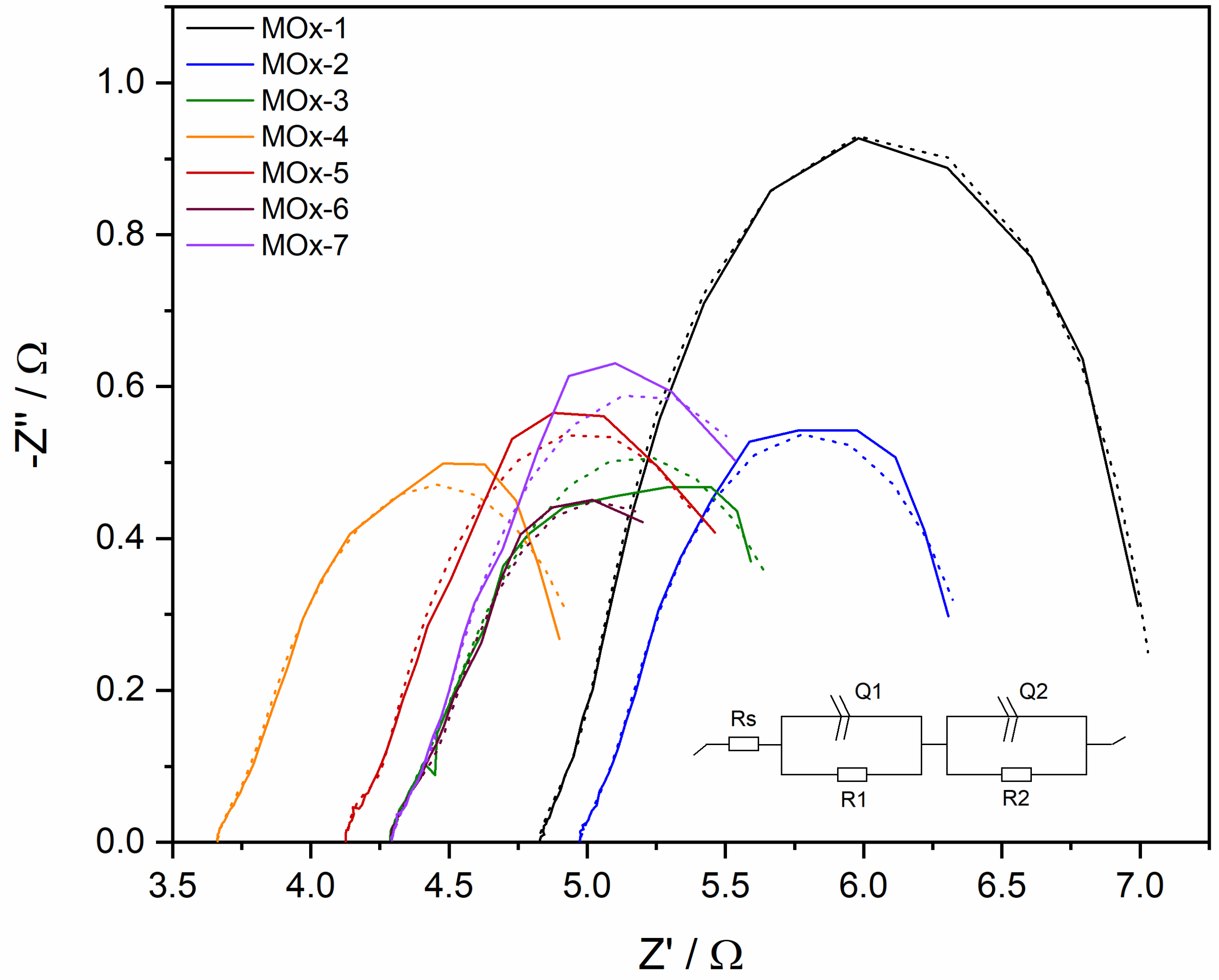

Figure S15. Nyquists plots obtained by PEIS at 10 mA cm^{-2} for MOx-*n* catalyst with the circuit employed to fit the experimental data. Experimental data and fit are represented as solid and dashed lines, respectively.

Table S3. Parameters obtained from fitting the Nyquists plots employing the circuit represented in Figure S14.

	MOx-1	MOx-2	MOx-3	MOx-4	MOx-5	MOx-6	MOx-7
**Rs**	4.8 ± 0.1	4.4 ± 0.5	4.8 ± 0.5	4.1 ± 0.5	3.9 ± 0.3	4.1 ± 0.2	4.3 ± 0.1
**R1**	0.2 ± 0.1	0.2 ± 0.1	0.2 ± 0.1	0.1 ± 0.1	0.1 ± 0.1	0.2 ± 0.1	0.2 ± 0.1
**R2**	2.1 ± 0.1	1.4 ± 0.1	1.5 ± 0.1	1.4 ± 0.5	1.4 ± 0.1	1.2 ± 0.1	1.6 ± 0.2

Table S4. Catalytic metric (geometric activity, reported stability, noble metal loading and Tafel slopes) of diverse catalysts towards the acidic OER. CF refers to carbon felt, CNT refers to carbon nanotubes, and CFs to carbon fibres.

Catalyst	Loading	η_{10} / mV	Stability test	Degradation rate	Tafel slope / mV dec^{-1}	Ref.
RuMnFeMoCo/CF	0.9 mg_{Ru} cm^{-2}	170	10 mA cm^{-2}, 1000 h	-	50	[1]
$RuIrFeCoCrO_2$	1 mg_{cat} cm^{-2}	185	10 mA cm^{-2}, 1000 h	n.r.	41	[2]
$RuIrFeCoNiO_2$	0.48 mg_{cat} cm^{-2}	189	10 mA cm^{-2}, 120 h	0.3 mV h^{-1}	49	[3]

https://doi.org/10.3390/xxxxx

RuNiMoCrFeOx/CNT	n.r.	219	100 mA cm^{-2}, 100 h	-	47	[4]
$(RuIrCrWCu)O_2$	0.25 mg_{cat} cm^{-2}	220	10 mA cm^{-2}, 12 h	-	54	[5]
$SrIrMnO_x$	0.5 mg_{cat} cm^{-2}	221	10 mA cm^{-2}, 1000 h	0.2 mV h^{-1} (on a PEMWE)	43	[6]
FeCoNiIrRu/CFs	2.45 wt.% $_{(Ir+Ru)}$	241	10 mA cm^{-2}, 12 h	-	153	[7]
AlAgAuCoCuFeIr-MoNiPdPtRhRu	0.25 mg_{cat} cm^{-2}	258	10 mA cm^{-2}, 11 h	9 mV h^{-1}	84	[8]
$(RuIrFeCoNi)O_2$	n.r.	261	CV 1.2-1.8 V, 3000 cycles	90% activity retention (10mA cm^{-2}, 24 h)	63	[9]
IrNiMnFeOx	0.4 mg_{Ir} cm^{-2}	279 ± 4	10 mA cm^{-2}, 150 h		43.8 ± 1.9	**This work**
IrNiMnFeCoOx	0.4 mg_{Ir} cm^{-2}	279 ± 4	10 mA cm^{-2}, 150 h		40.9 ± 0.5	**This work**
IrFeCoNiCu	0.29 mg_{Ir} cm^{-2}	302	10 mA cm^{-2}, 12 h	-	58	[10]
IrO_x-Co_3O_4	0.12 mg_{cat} cm^{-2}	316	10 mA cm^{-2}, 1000 h	0.05 mV h^{-1}	52	[11]
IrOx/CFs	0.4 mg_{Ir} cm^{-2}	333 ± 9	10 mA cm^{-2}, 2 h	n.r.	51	[12]

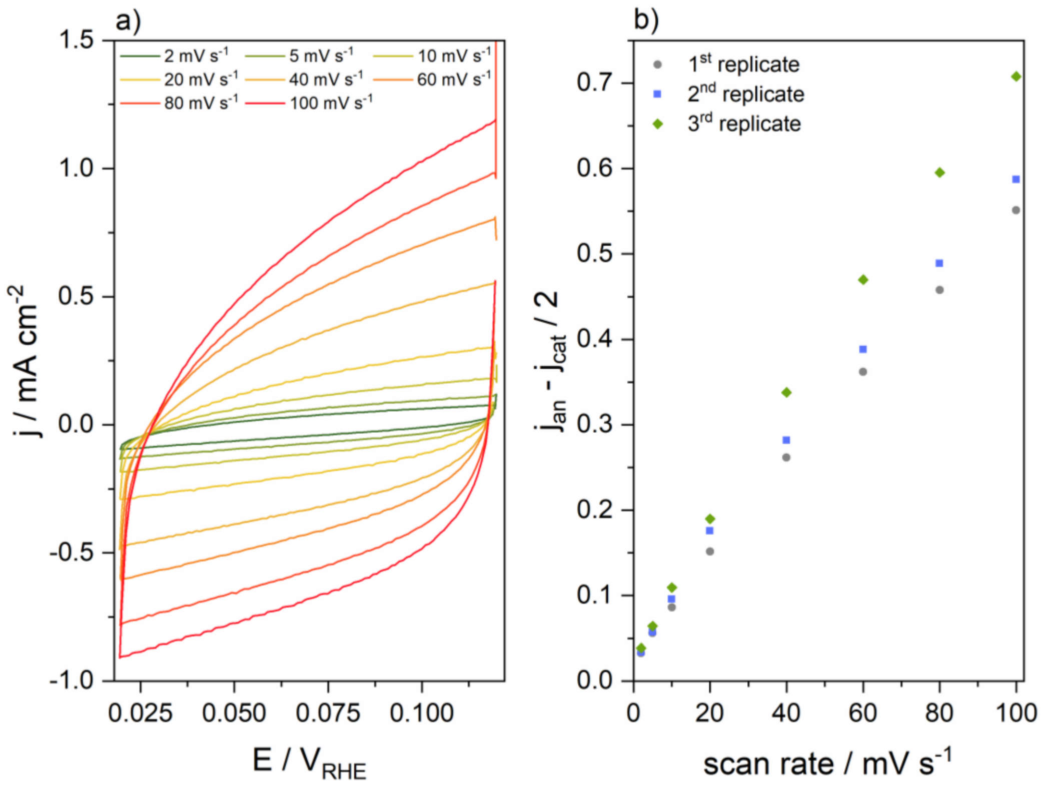

Figure S16. (**a**) CV performed on a non-Faradaic region for MOx-4 and (**b**) derived double layer capacitance (C_{DL}) for the three replicates.

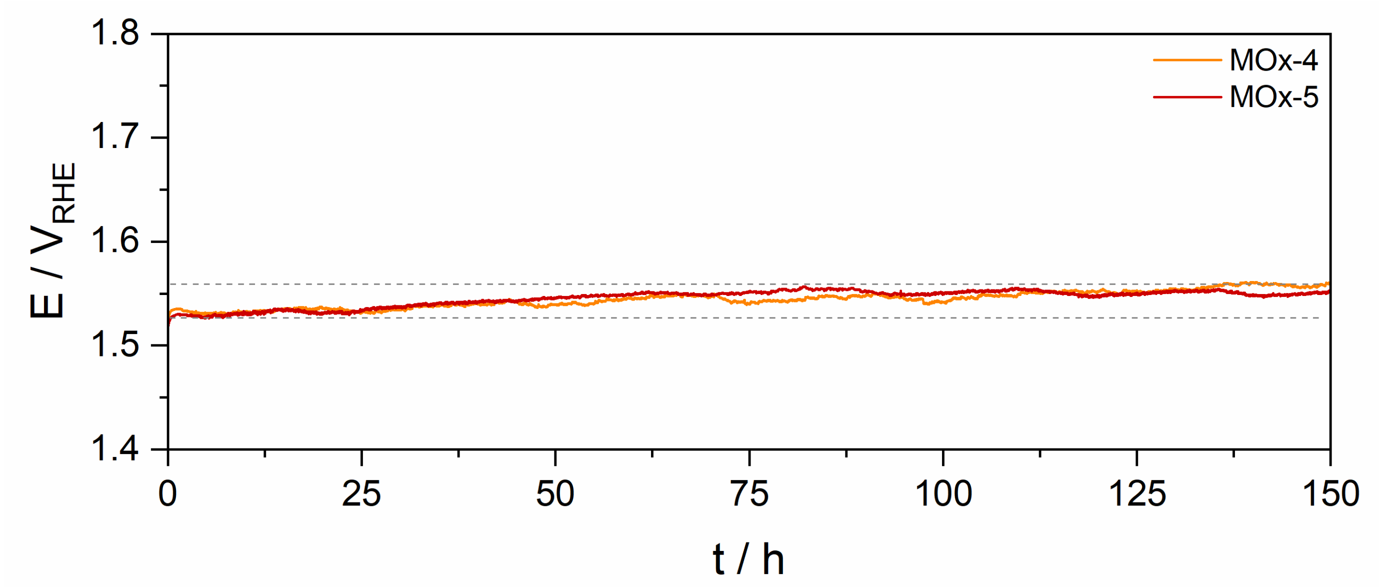

Figure S17. Long-term stability test performed by CP (10 mA cm^{-2}, 150 h) for MOx-4 and MOx-5.

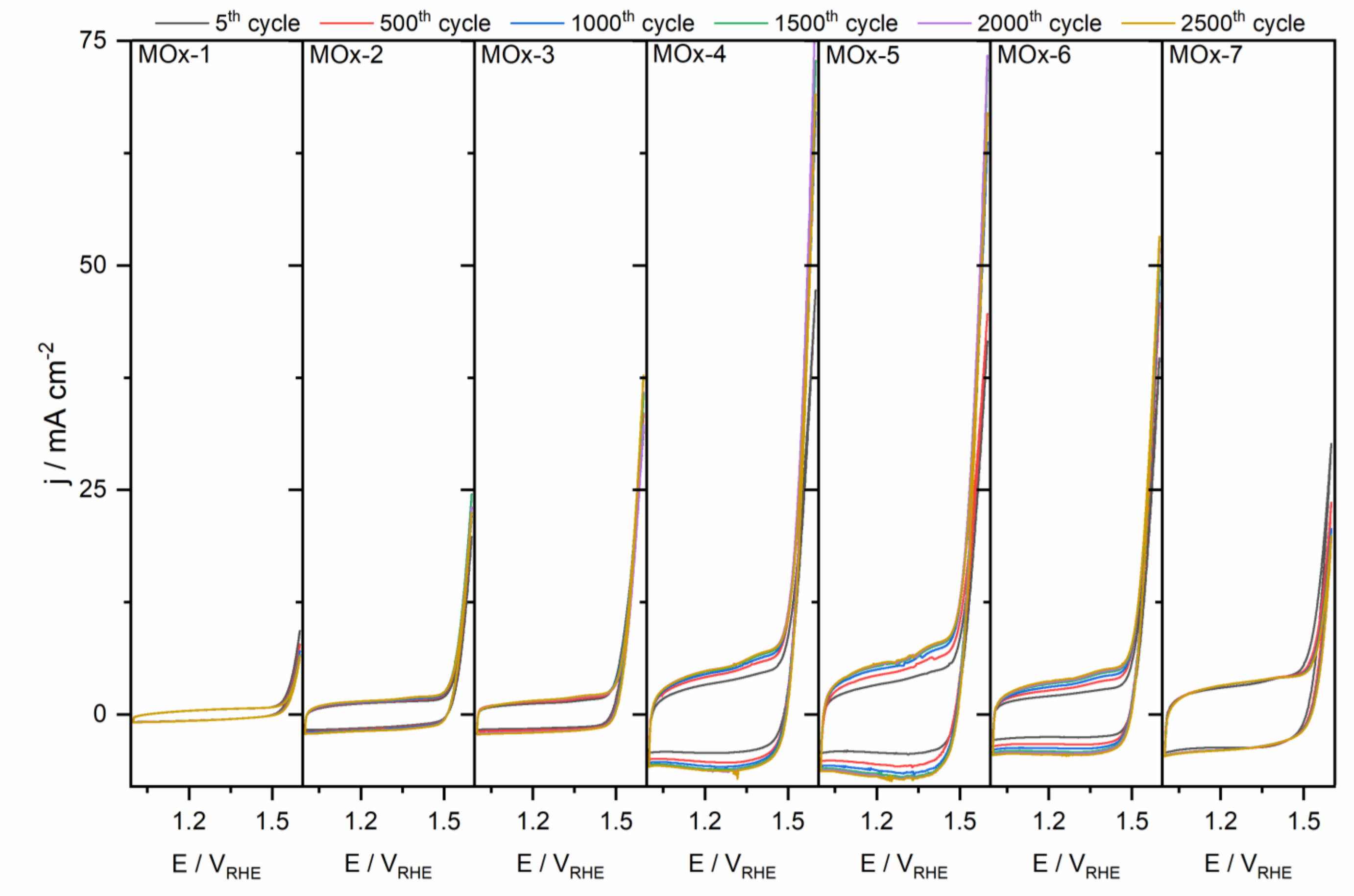

Figure S18. Stability assessed under dynamic conditions by CV (1.0-1.6 V_{RHE}, 50 mV s^{-1}, 2500 cycles).

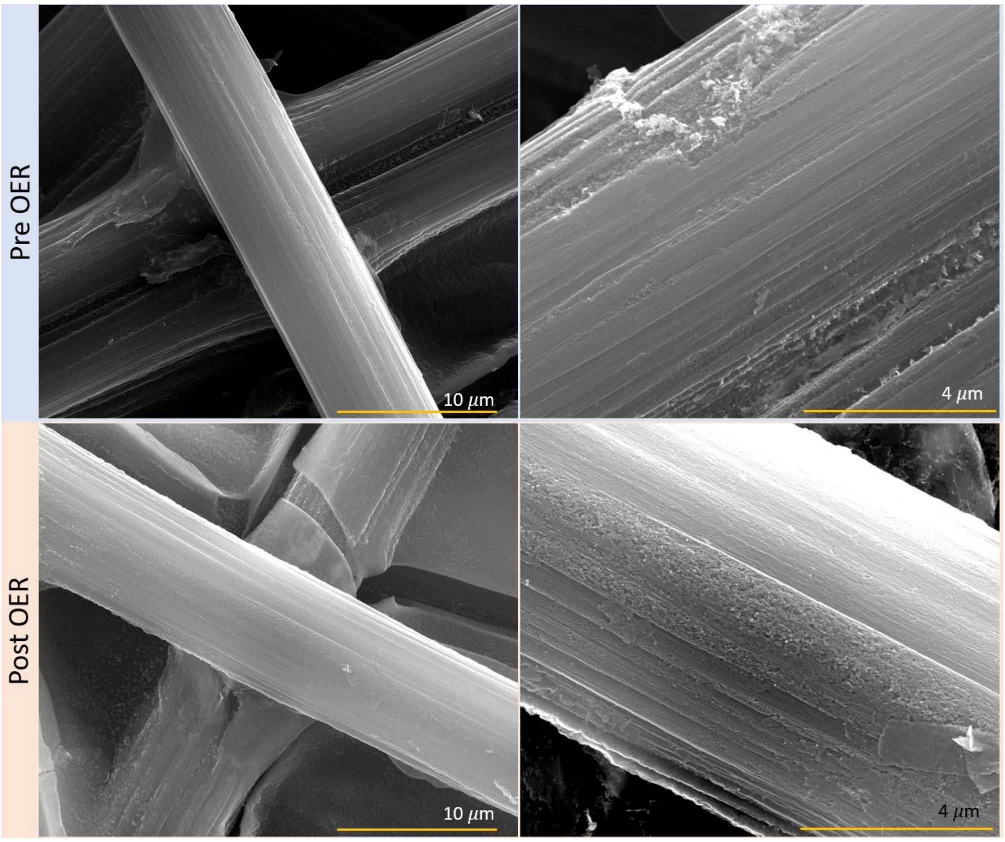

Figure S19. SEM-EDS images for MOx-1 prior and after OER.

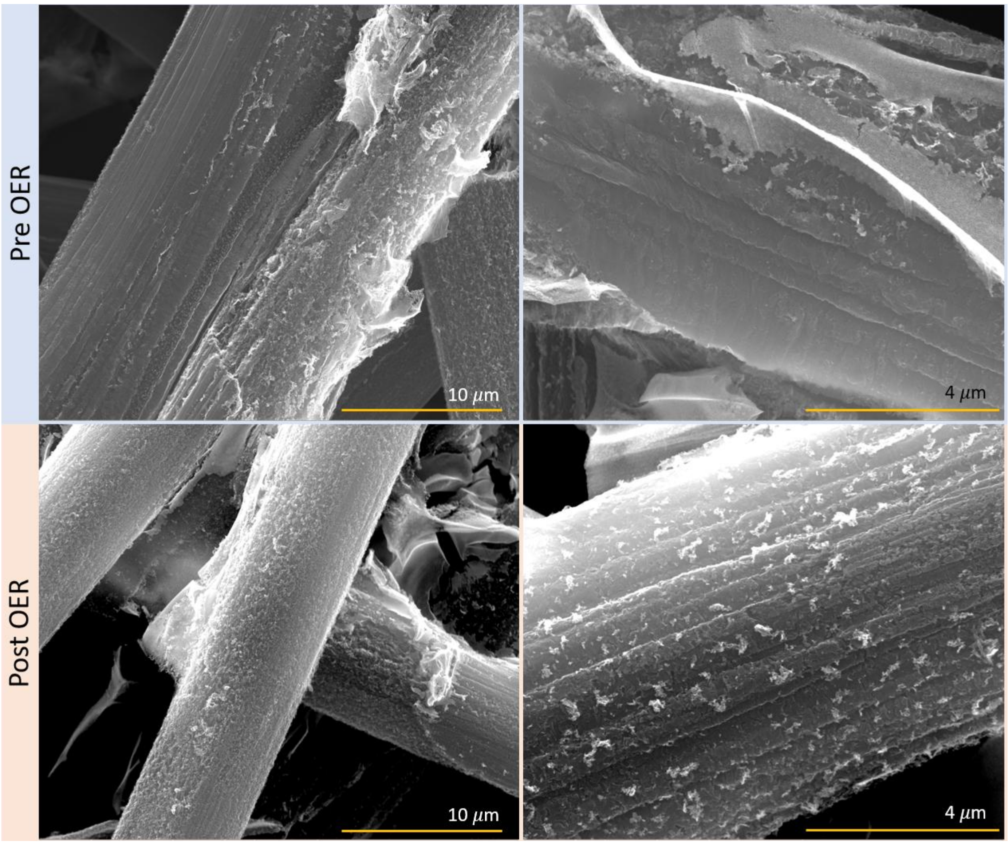

Figure S20. SEM-EDS images for MOx-2 prior and after OER.

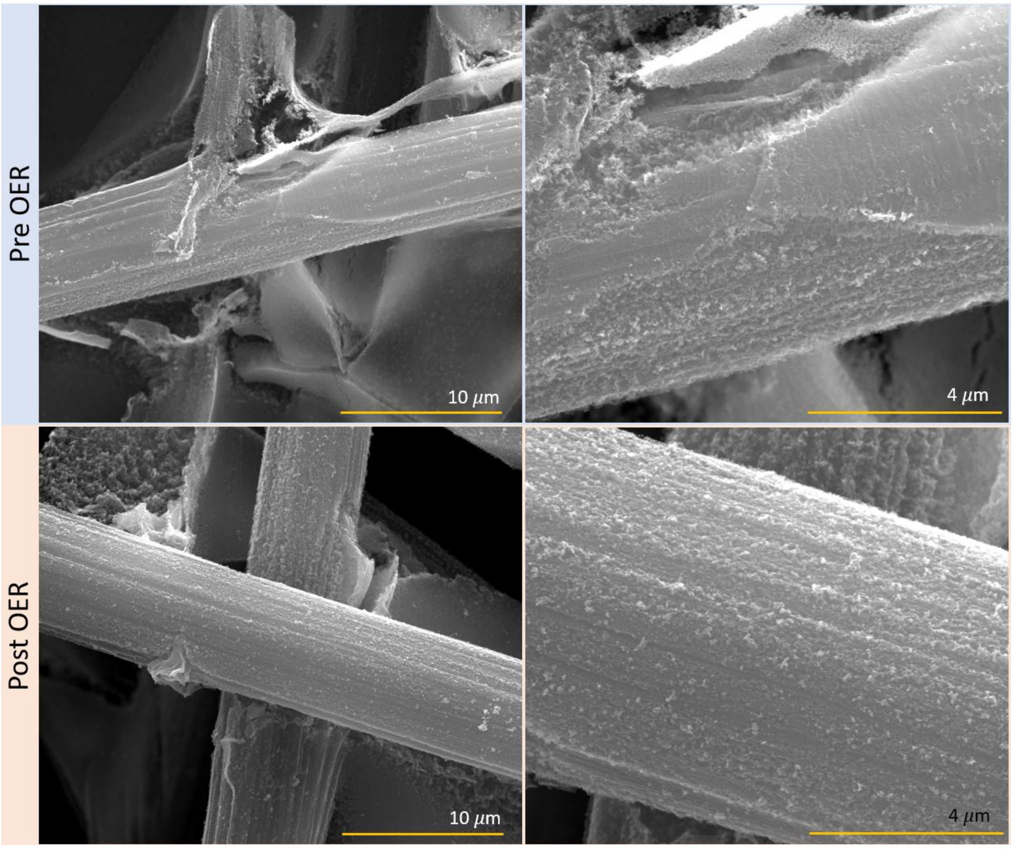

Figure S21. SEM-EDS images for MOx-3 prior and after OER.

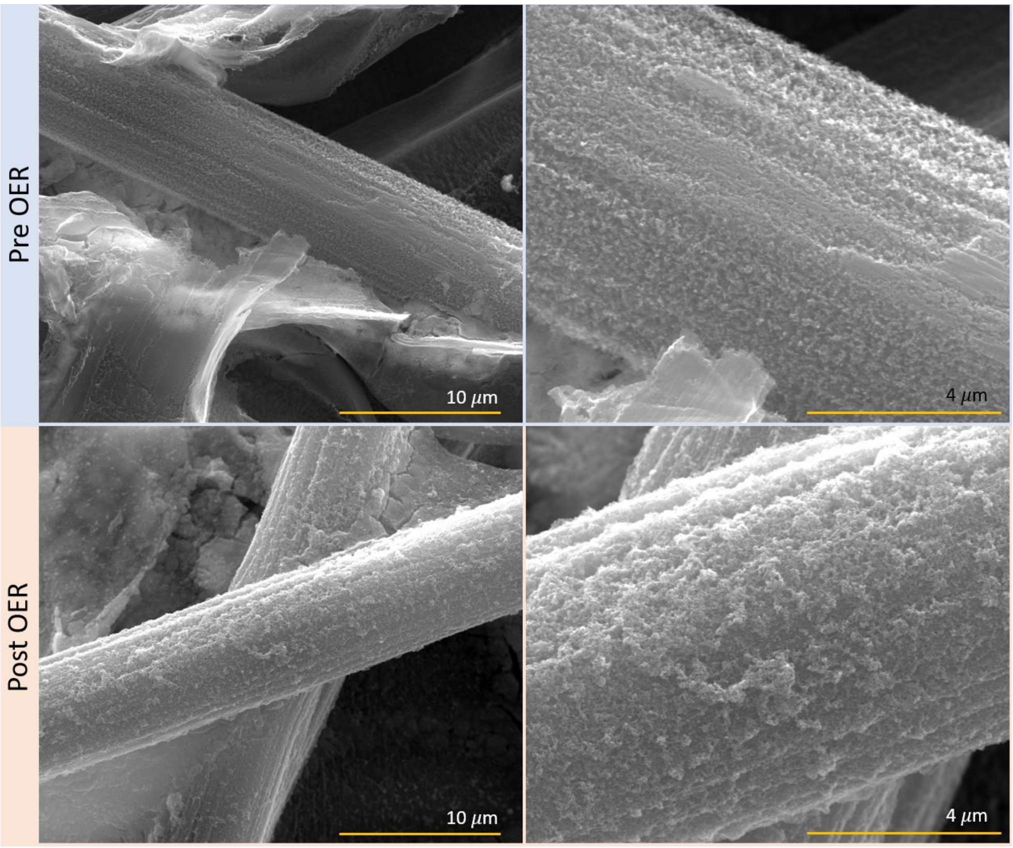

Figure S22. SEM-EDS images for MOx-4 prior and after OER.

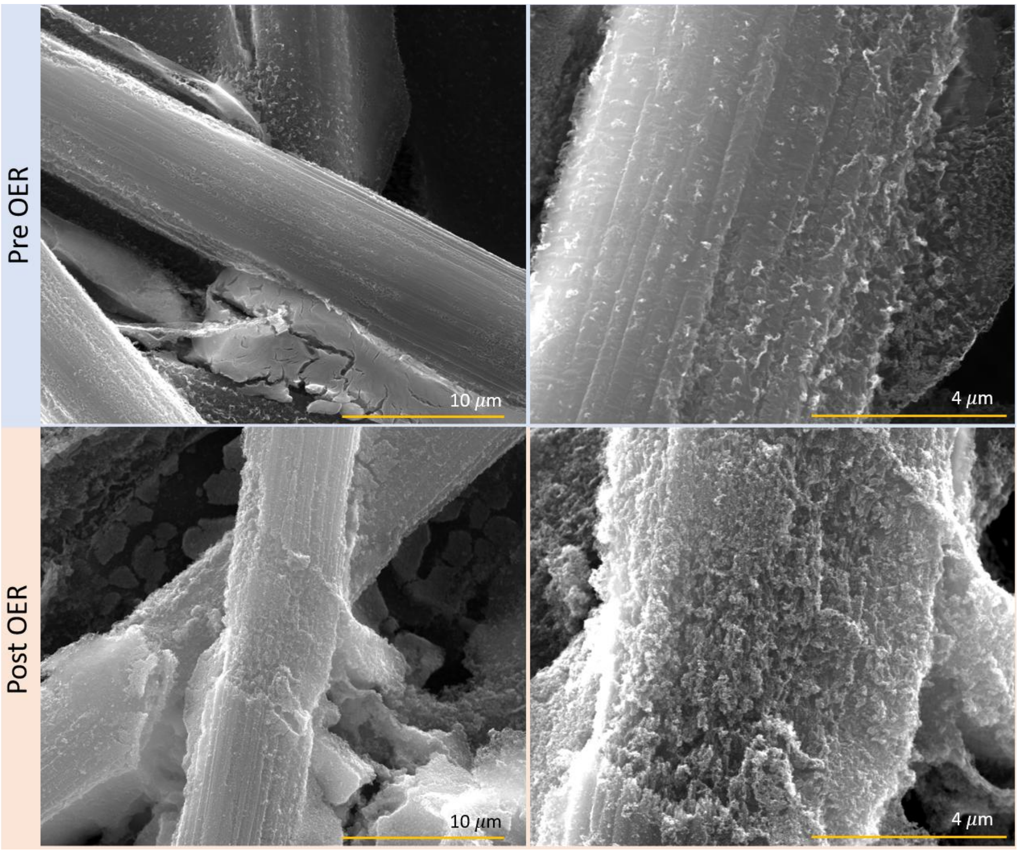

Figure S23. SEM-EDS images for MOx-5 prior and after OER.

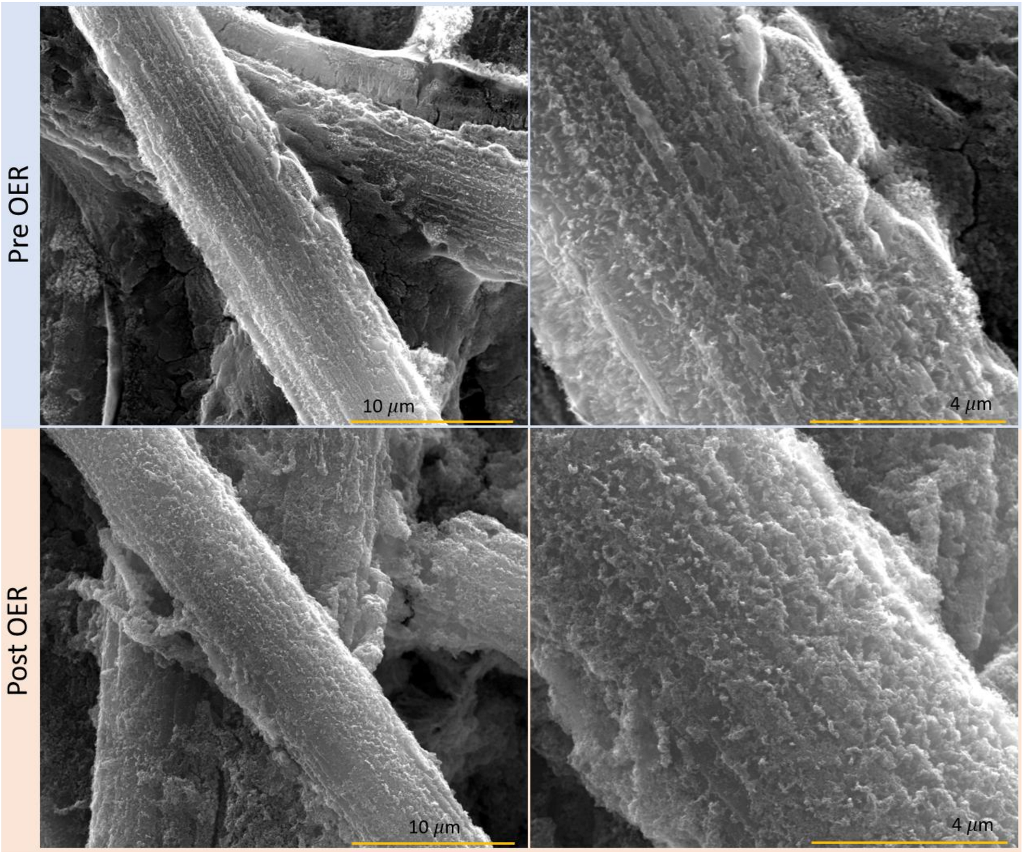

Figure S24. SEM-EDS images for MOx-6 prior and after OER.

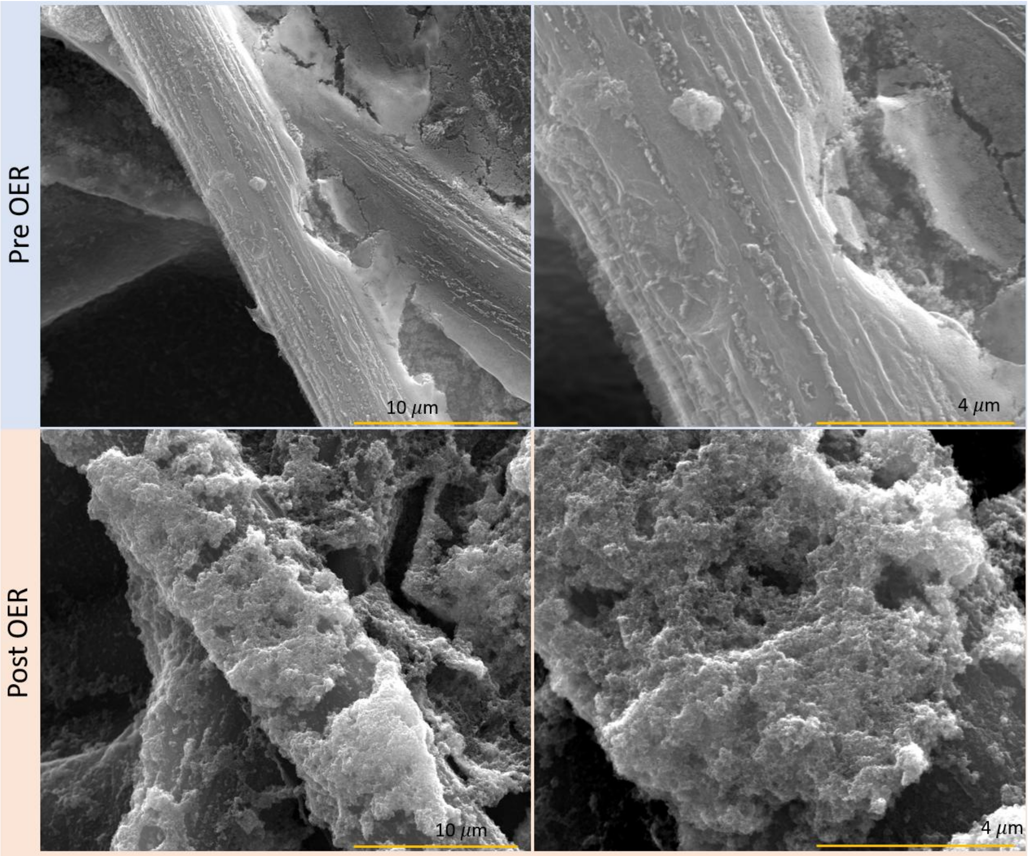

Figure S25. SEM-EDS images for MOx-7 prior and after OER.

References

1. Chen, J.; Ma, J.; Huang, T.; Liu, Q.; Liu, X.; Luo, R.; Xu, J.; Wang, X.; Jiang, T.; Liu, H.; et al. Iridium-Free High-Entropy Alloy for Acidic Water Oxidation at High Current Densities. *Angew. Chem. Int. Ed.* **2025**, *64*, e202503330. https://doi.org/10.1002/anie.202503330.
2. T. Zhang, Q. Liu, H. Bao, M. Wang, N. Wang, B. Zhang, H.J. Fan, Atomically thin high-entropy oxides via naked metal ion self-assembly for proton exchange membrane electrolysis, Nat Commun, 16 (2025) 1037.
3. Hu, C.; Yue, K.; Han, J.; Liu, X.; Liu, L.; Liu, Q.; Kong, Q.; Pao, C.-W.; Hu, Z.; Suenaga, K.; et al. Misoriented high-entropy iridium ruthenium oxide for acidic water splitting. *Sci. Adv.* **2023**, *9*, eadf9144. https://doi.org/10.1126/sciadv.adf9144.
4. Yu, Y.; Li, H.; Liu, J.; Xu, W.; Zhang, D.; Xiong, J.; Li, B.; Omelchuk, A. O.; Lai, J.; Wang, L. High entropy stabilizing lattice oxygen participation of Ru-based oxides in acidic water oxidation. *J. Mater. Chem. A* **2022**, *10*, 21260–21265. http://dx.doi.org/10.1039/D2TA06128G.
5. Miao, X.; Peng, Z.; Shi, L.; Zhou, S. Insulating High-Entropy Ruthenium Oxide as a Highly Efficient Oxygen-Evolving Electrocatalyst in Acid. *ACS Catal.* **2023**, 13, 6, 3983-3989. https://doi.org/10.1021/acscatal.2c06276.
6. Zhao, J. W.; Yue, K.; Pei, Z.; Yang, J.; Luan, D.; Lou, X. W. Highly Active IrO_x Formation on Strontium Manganite for Acidic Oxygen Evolution. *Adv. Mater.* **2026**, *38*, e15749. https://doi.org/10.1002/adma.202515749.
7. Zhu, H.; Zhu, Z.; Hao, J.; Sun, S.; Lu, S.; Wang, C.; Ma, P.; Dong, W.; Du, M. High-entropy alloy stabilized active Ir for highly efficient acidic oxygen evolution. *Chem. Eng. J.* **2021**, *431*, 133251. https://doi.org/10.1016/j.cej.2021.133251.
8. Cai, Z.X.; Goou, H.; Ito, Y.; Tokunaga, T.; Miyauchi, M.; Abe, H.; Fujita, T. Nanoporous ultra-high-entropy alloys containing fourteen elements for water splitting electrocatalysis. *Chem. Sci.* **2021**, *12*, 11306–11315. https://doi.org/10.1039/D1SC01981C.
9. Li, L.; Wang, M.; Feng, D.; Zhang, Y.; Ren, H.; Kang, P. Reducing Noble Metal Content in Water Electrolysis Catalysts: A Study on High-Entropy Oxides. *J. Alloys Compd.* **2024**, *1009*, 176947. https://doi.org/10.1016/j.jallcom.2024.176947.
10. Maulana, A.L.; Chen, P.C.; Shi, Z.; Yang, Y.; Lizandara-Pueyo, C.; Seeler, F.; Abruña, H.D.; Muller, D.; Schierle-Arndt, K.; Yang, P. Understanding the Structural Evolution of IrFeCoNiCu High-Entropy Alloy Nanoparticles under the Acidic Oxygen Evolution Reaction. *Nano Lett.* **2023**, *23*, 6637–6644. https://doi.org/10.1021/acs.nanolett.3c01831.

11. Li, G.; Priyadarsini, A.; Xie, Z.; Kang, S.; Liu, Y.; Chen, X.; Kattel, S.; Chen, J. G. Achieving Higher Activity of Acidic Oxygen Evolution Reaction Using an Atomically Thin Layer of IrO_x over Co_3O_4. *J. Am. Chem. Soc*. **2025**, *147*, 7008–7016. https://doi.org/10.1021/jacs.4c17915.
12. Riera, S.R.; Pérez-Mas, A.M.; Bes, R.; González de la Vega, M.; González-Ingelmo, M.; Sierra Gómez, U.A.; Blanco, C.; Santamaría, R.; Rocha, V.G.; Esquius, J.R. Self-Supported $IrMnFeCoNiO_x$ High Entropy Spinel as Acid Resistant and Active Oxygen Evolution Catalyst. *Mater. Today Sustain.* **2026**, *33*, 101293. https://doi.org/10.1016/j.mtsust.2025.101293.

Disclaimer/Publisher's Note: The statements, opinions and data contained in all publications are solely those of the individual author(s) and contributor(s) and not of MDPI and/or the editor(s). MDPI and/or the editor(s) disclaim responsibility for any injury to people or property resulting from any ideas, methods, instructions or products referred to in the content.
